# Critical Role of Flow Cytometric Immunophenotyping in the Diagnosis, Subtyping, and Staging of T-Cell/NK-Cell Non-Hodgkin’s Lymphoma in Real-World Practice: A Study of 232 Cases From a Tertiary Cancer Center in India

**DOI:** 10.3389/fonc.2022.779230

**Published:** 2022-03-01

**Authors:** Prashant R. Tembhare, Gaurav Chatterjee, Anumeha Chaturvedi, Niharika Dasgupta, Twinkle Khanka, Shefali Verma, Sitaram G. Ghogale, Nilesh Deshpande, Karishma Girase, Manju Sengar, Bhausaheb Bagal, Hasmukh Jain, Dhanalaxmi Shetty, Sweta Rajpal, Nikhil Patkar, Tushar Agrawal, Sridhar Epari, Tanuja Shet, Papagudi G. Subramanian, Sumeet Gujral

**Affiliations:** ^1^ Hematopathology Laboratory, Advanced Centre for Treatment, Research and Education in Cancer (ACTREC), Tata Memorial Center, Homi Bhabha National Institute (HBNI) University, Mumbai, India; ^2^ Department of Medical Oncology, Tata Memorial Center, HBNI University, Mumbai, India; ^3^ Department of Cancer Cytogenetics, ACTREC, Tata Memorial Center, HBNI University, Mumbai, India; ^4^ Department of Pathology, Tata Memorial Center, HBNI University, Mumbai, India

**Keywords:** immunophenotyping, T cell, non-Hodgkin’s lymphoma, real-world practice, flow cytometry

## Abstract

**Background:**

T-cell/NK-cell non-Hodgkin’s lymphoma (T/NK-NHL) is an uncommon heterogeneous group of diseases. The current classification of T/NK-NHL is mainly based on histopathology and immunohistochemistry. In practice, however, the lack of unique histopathological patterns, overlapping cytomorphology, immunophenotypic complexity, inadequate panels, and diverse clinical presentations pose a great challenge. Flow cytometric immunophenotyping (FCI) is a gold standard for the diagnosis, subtyping, and monitoring of many hematological neoplasms. However, studies emphasizing the role of FCI in the diagnosis and staging of T/NK-NHL in real-world practice are scarce.

**Methods:**

We included T-cell non-Hodgkin’s lymphoma (T-NHL) patients evaluated for the diagnosis and/or staging of T/NK-NHL using FCI between 2014 and 2020. We studied the utility of FCI in the diagnosis and subtyping of T/NK-NHL and correlated the FCI findings with the results of histopathology/immunohistochemistry. For correlation purposes, patients were categorized under definitive diagnosis and subtyping, inadequate subtyping, inadequate diagnosis, and misdiagnosis based on the findings of each technique.

**Results:**

A total of 232 patients were diagnosed with T/NK-NHL. FCI findings provided definitive diagnoses in 198 patients and subtyping in 187/198 (95.45%) patients. The correlation between FCI and histopathological/immunohistochemistry results (n = 150) demonstrated an agreement on the diagnosis and subtyping in 69/150 (46%) patients. Of the remaining cases, the diagnosis and subtyping were inadequate in 64/150 (42.7%), and 14/150 (9.33%) were misdiagnosed on histopathology/immunohistochemistry results. FCI provided definitive diagnosis and subtyping in 51/64 (79.7%) patients. Among these, 13 patients diagnosed with peripheral T-cell lymphoma not-otherwise-specified were reclassified (angioimmunoblastic T-cell lymphoma (AITL)-11 and prolymphocytic leukemia-2) on FCI. It corrected the diagnosis in 14 patients that were misdiagnosed (6 B-cell NHL (B-NHL), 3 Hodgkin’s lymphoma, 1 acute leukemia, and 1 subcutaneous panniculitis-like T-cell lymphoma) and misclassified (3 T-NHL) on histopathological results. AITL was the commonest T-NHL misclassified on histopathological results. FCI also confirmed the definite involvement in 7/83 (8.4%) and 27/83 (32.5%) bone marrow (BM) samples reported as suspicious and uninvolved, respectively, on histopathological evaluation.

**Conclusion:**

AITL was the most frequently diagnosed T/NK-NHL in this study. FCI provided a distinct advantage in detecting BM involvement by T/NK-NHL, especially in patients with low-level involvement. Overall, our study concluded that FCI plays a critical role in the diagnosis, subtyping, and staging of T/NK-NHL in real-world practice.

## 1 Introduction

T-cell non-Hodgkin’s lymphoma (T-NHL) is a heterogeneous group of aggressive NHL arising from T-cell and NK-cell subsets accounting for approximately 10%–15% of all NHLs (1–3). The prevalence of T-NHL is slightly higher in Asia and Central-South America than in Western countries (2). The current WHO classification of hematopoietic neoplasms has enlisted more than 30 definite or provisional entities under the heading of mature T- and NK-cell neoplasms (3). The diagnosis and subtyping of T-cell/NK-cell NHL (T/NK-NHL) heavily rely on a multifactorial approach that includes clinical presentation, morphology, immunophenotype, and chromosomal abnormalities (4). In recent years, the treatment regimens for T-NHL has seen great improvements with the availability of newer agents such as denileukin diftitox, brentuximab vedotin, pralatrexate, alemtuzumab, vorinostat, and romidepsin (5–8). The selection of newer therapeutic agents including targeted therapy (e.g., brentuximab vedotin and alemtuzumab) may be subtype specific (7, 9, 10). Further, in middle- or low-income countries with limited resources, the prognosis of lymphoma, which can vary between the subtypes of T/NK-NHL, can help in prioritizing the available resources (11, 12). Thus, accurate diagnosis and subtyping of T/NK-NHL has become a basic requirement for the clinical management of patients.

In practice, however, lack of unique histopathological patterns, overlapping cytomorphological features, immunophenotypic complexity, paucity of specific genetic abnormalities, and diverse clinical presentations pose a great challenge to the diagnosis and subtyping in the majority of mature T/NK-NHLs (12–17). Despite recent technical advances, approximately 30% of peripheral T-NHL cases remain unclassifiable and categorized as “not otherwise specified” (4, 15). Additionally, limited resources, inadequate tissue, lack of expertise, and financial constraints add up to the variability in the diagnostic workup, accounting for relatively poor reproducibility of the diagnoses in T/NK-NHL (18–22).

In real-world practice, most of the centers rely on histopathological findings and minimal immunohistochemistry (IHC)-based immunophenotype for the lymphoma diagnosis and classification (18, 22–24). For the definitive diagnosis, an optimal IHC-based immunophenotyping workup is required, which depends upon the tissue adequacy and the availability of a comprehensive antibody panel. Furthermore, obtaining surgical excisional biopsies (SEBs) in patients with the involvement of deep-seated lymph nodes and extranodal sites is practically improbable in many instances. In such a scenario, the diagnosis solely depends on the core-needle biopsies (CNBs) and radiological findings (25, 26). Several reports have highlighted the limitations of CNB-based diagnosis of NHL due to inadequate tissue as being responsible for the inability to evaluate histopathological patterns and immunophenotype by IHC (27–32). Thus, lower incidence, inadequate immunophenotyping workup, limited tissue, and lack of expertise collectively contribute to inadequate opinion, incorrect subtyping, or sometimes even misdiagnosis such as Hodgkin’s lymphoma, B-cell NHL (B-NHL), inflammatory process, or reactive proliferation in a significant percentage of T-NHL, skewing the true incidence (12, 14, 25, 33–51).

Flow cytometric immunophenotyping (FCI) is a powerful tool for single-cell analysis that allows the study of multiple protein expressions simultaneously in thousands to millions of cells in a short duration of time (52). It is widely available and routinely used for the diagnosis, subtyping, staging, and monitoring of hematological neoplasms like acute leukemia (AL), myelodysplastic syndrome, B-NHL, and multiple myeloma (53). The unique ability of advanced flow cytometry instruments in simultaneous detection ≥8 proteins on a single cell and the availability of an expanding list of new antibodies and fluorochromes have made it possible to trace the cells of tumor origin easily (52, 54, 55). Many studies have documented the role of FCI in the diagnosis or exclusion of T-NHL describing immunophenotypic profile and clonality assessment (14, 26, 44, 52, 56–65). However, studies emphasizing the role of FCI in the diagnosis and staging of T/NK-NHL in real-world practice are scarce. This study highlights the critical contribution of FCI-based immunophenotyping and clonality assessment in diagnosing and staging T/NK-NHL in routine practice.

## 2 Materials and Methods

**Figure 1:**
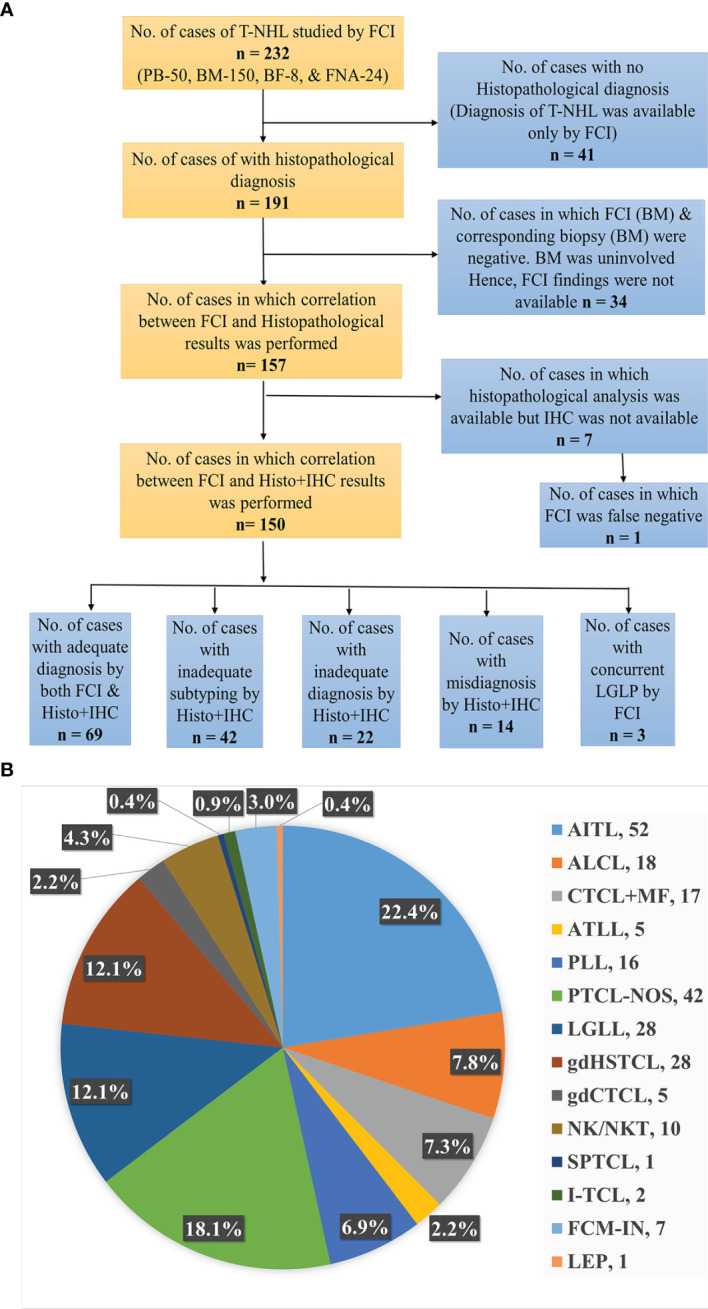
**(A)** Flowchart representing the distribution of T-cell/NK-cell NHL patients studied. BM, bone marrow; BF, body fluids; FCI, flow cytometric immunophenotyping; FNA, fine-needle aspiration; Histo, histopathology; IHC, immunohistochemistry; PB, peripheral blood; NHL, non-Hodgkin’s lymphoma. **(B)** Distribution of subtypes of T-cell/NK-cell NHL patients. AITL, angioimmunoblastic T-cell lymphoma; ALCL, anaplastic large T-cell lymphoma; ATLL, adult T-cell leukemia/lymphoma; CTCL, cutaneous T-cell lymphoma; FCM, flow cytometry; FHTCL, follicular helper type T-cell lymphoma; HSTCL, hepatosplenic T-cell NHL; I, intestinal; IN, inadequate; LEP, lupus erythematosus panniculitis; LGLL, large granular lymphocytic leukemia; M, male; MF, mycosis fungoides; NK/NKTCL, NK-/T-cell lymphoma; PTCL-NOS, peripheral T-cell lymphoma not otherwise specified; PLT, platelets; PLL, prolymphocytic leukemia; SPTCL, subcutaneous panniculitis-like T-cell lymphoma.

This study was approved by the Institutional Ethical Committee. Patients diagnosed with mature T-/NK-cell neoplasms were identified from the electronic medical records (EMRs) of Tata Memorial Center (TMC), India. We included the patients evaluated for FCI in Hematopathology Laboratory for either diagnosis or staging of the T/NK-NHL (**Figure 1**). This retrospective study included patients investigated for the last 7 years (2014–2020). The clinical details, laboratory findings, and treatment history were recorded from the EMR. The final diagnosis and subtyping were made in accordance with WHO 2008 and 2016 hematolymphoid classification based on the available details on clinical presentation, cytomorphological and histopathological features, immunophenotypic (using both IHC and FCI) data, radiological features, and genetic findings (66, 67).

### 2.1 Cytomorphology

Peripheral blood (PB), bone marrow (BM) aspiration, body fluid (BF), and fine-needle aspiration (FNA) smears were stained with Wright’s stain. Morphological details, including the adequacy of cellularity, differential count, and nuclear and cytoplasmic details of lymphocytes, were studied. Tissue biopsy (TB) examination was conducted on H&E-stained sections in conjunction with IHC studies. TB sections were performed using 5-µm-thick, formalin-fixed, paraffin-embedded tissue, and IHC was performed after heat-induced epitope retrieval as described below.

### 2.2 Immunohistochemistry Studies

IHC was performed using the avidin–biotin complex method on formalin-fixed paraffin sections using automated immunostainers (Roche Diagnostics, Skopje, North Macedonia: Ventana BenchMark XT). The panel of antibodies studied included LCA, CD1a, CD3, CD5, CD4, CD7, CD8, CD10, CD15, CD20, CD23, CD30, CD31, CD34, CD43, CD56, CD57, CD138, CD163, AE1/AE3, ALK-1, BCL2, BCL6, BOB1, c-Kit, C-myc, CK7, CK20, EMA, Granzyme-B, MIB1, myeloperoxidase (MPO), MUM-1, OCT2, PAX-5, terminal deoxynucleotidyl transferase (TdT), and EBVLMP1. The details of antibody dilution, clone, and company are given in **Supplementary Table S1**. EBER-ISH was done with an EBV probe *in situ* hybridization (ISH) kit (Novocastra Laboratories Ltd., Newcastle upon Tyne, UK). The IHC panels performed were mainly based on the tissue adequacy, availability of reagents at the time of evaluation, and judgment of the reporting pathologist. It was categorized into inadequate and adequate IHC panels. Based on the “International Peripheral T-Cell and Natural Killer/T-Cell Lymphoma Study 2008,” the IHC panel with ≤5 markers including only basic markers such as LCA, CD3, CD5, CD20, and PAX-5 were categorized as inadequate IHC panel and the IHC panel with >5 markers including additional markers useful for T-NHL diagnosis and classification such as CD4, CD7, CD8, CD10, CD23, CD30, CD34, CD56, CD57, ALK-1, BCL6, C-myc, Granzyme-B, MIB1, TdT, and EBVLMP1 were categorized as adequate IHC panel (2). However, IHC for PD1, CXCL13, ICOS, βF1, and TCRδ1 was not available in most of the samples.

### 2.3 Flow Cytometric Immunophenotyping

**Figure 2:**
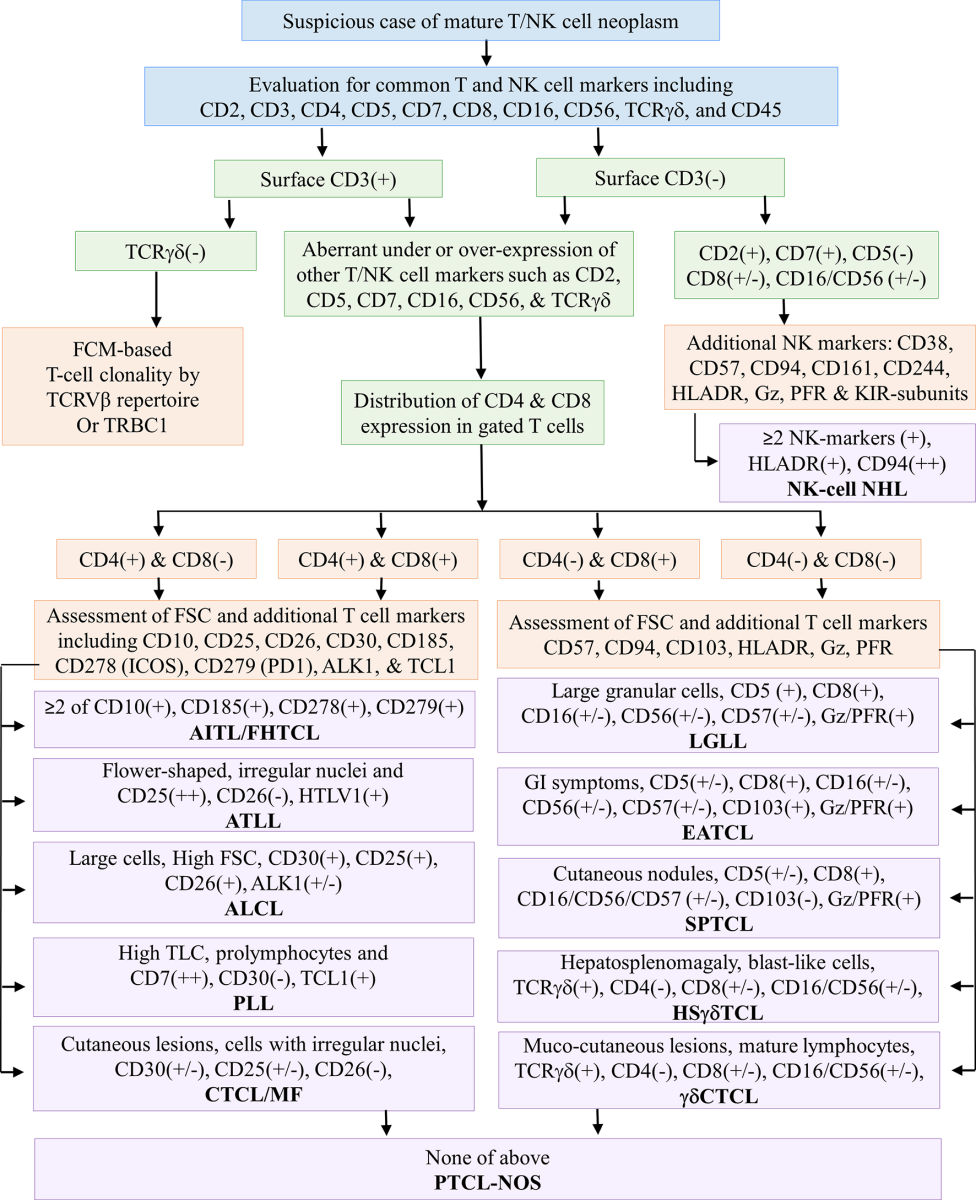
Flowchart demonstrates an immunophenotypic approach for assessing common T-cell markers using a primary antibody panel followed by the selection of an additional antibody panel based on the CD4 and/or CD8 distribution. The latter half of the flowchart demonstrates the utility of selective markers for the subtyping of T-cell NHL. AITL, angioimmunoblastic T-cell lymphoma; ALCL, anaplastic large T-cell lymphoma; ATLL, adult T-cell leukemia/lymphoma; CTCL, cutaneous T-cell lymphoma; EATCL, enteropathy-associated T-cell lymphoma; FCM, flow cytometry; FHTCL, follicular helper type T-cell lymphoma; Gz, granzyme; HSTCL, hepatosplenic T-cell NHL; I, intestinal; IN, inadequate; KIR, killer immunoglobulin-like receptors; LEP, lupus erythematosus panniculitis; LGLL, large granular lymphocytic leukemia; M, male; MF, mycosis fungoides; NK/NKTCL, NK-/T-cell lymphoma; PFR, perforin; PTCL-NOS, peripheral T-cell lymphoma not otherwise specified; PLT, platelets; PLL, prolymphocytic leukemia; SPTCL, subcutaneous panniculitis-like T-cell lymphoma; (+) positive expression; (++) strong positive expression; (+/−) positive or negative or heterogeneous expression; (−) negative expression.

FCI was performed using the bulk–lyse–wash technique as described elsewhere (68–70). In brief, the cell suspension was prepared by erythrocyte lysing with ammonium chloride-based lysing reagent (100 µl to 2 ml of sample in 15 to 48 ml of lysing reagent). The cells were stained a 10- to 13-color comprehensive antibody panel (**Supplementary Table S2**) and acquired on Navios and CytoFlex flow cytometry instruments (Beckman Coulter, Miami, FL, USA). For each panel, the following were acquired: in samples with ≥10% atypical lymphocyte on microscopic examination, a minimum of 100,000 events; in samples with <10% atypical lymphocyte, a minimum of 500,000 events. Initially, we studied a primary antibody panel (**Supplementary Table S2A**) that included antibodies against CD2, CD3, CD4, CD5, CD7, CD8, CD10, CD11c, CD16, CD19, CD20, CD23, CD26, CD38, CD45, CD49d, CD56, CD200, kappa/lambda, and γδT-cell receptor (TCRγδ). Based on the expressions of CD4 and CD8 in tumor cells from the results of the primary antibody panel, additional antibody panels (**Supplementary Table S2B**) were performed (**Figure 2**). T-cell clonality was studied using TCR-Vβ staining using IOTest Beta Mark TCR Repertoire Kit (Beckman Coulter, Marseille, France) that includes monoclonal antibodies (mAbs) against 24 distinct TCR-Vβ families (**Supplementary Table S2C**) (62). T-cell clonality was also studied by TRBC1 (**Supplementary Table S2D**) expression in a few recent cases (71). It was assessed in those samples only where suspicious T cells were surface CD3 positive and TCRγδ negative. A cutoff of the cluster of a minimum of 50 events of abnormal cells was used to define the abnormal T-cell population. Data were analyzed with KaluzaV2.1 software (Beckman Coulter, Miami, FL, USA) using predesigned templates.

#### 2.3.1 Gating Strategy

The gating strategy is shown in **Supplementary Figure S1**. Initial gating was based on CD45 versus side scatter (SSC) characteristics in which lymphoid cells were gated using strong CD45 and low SSC followed by an evaluation of T cells using CD2, CD3, CD4, CD5, CD7, and CD8 expressions. In some cases, an alternative gating strategy was also implemented using CD45 or SSC versus pan-T-cell markers such as CD2, CD3, CD5, and CD7. T cells were studied for expression patterns (under or over or asynchronous expression) of pan-T-cell markers such as CD2, CD3, CD5, and CD7 as well as NK-cell markers such as CD16 and CD56 followed by CD4 and CD8 restriction. Based on the CD4 or CD8 expression, additional markers were studied. As demonstrated in **Figure 2**, in samples with predominantly CD4-expressing abnormal T cells, the gated T-cell population was further studied for CD10, CD25, CD26, CD30, CD185 (CXCR5), CD278 (ICOS), CD279 (PD1), ALK-1, and TCL1. In samples with predominantly CD8-expressing abnormal T cells, the gated T-cell population was further studied for CD16, CD25, CD26, CD30, CD56, CD57, CD94, CD161, CD244, granzyme, and perforin. The immunophenotypic approach adapted to diagnose and subclassify T-NHL or NK-NHL is shown in **Figure 2**.

For T-cell clonality assessment, the initial gating strategy was based on CD45 vs. CD3 expression followed by CD4+CD3+ T and CD8+CD3+ T-cell population. The suspicious T-cell population was further isolated using the altered expressions in CD4, CD3, CD5, CD7, CD16, and CD56. The clonality was defined as direct or indirect as described earlier (62, 71–73).

### 2.4 Cytogenetic Studies

The cytogenetic evaluation was performed using conventional karyotyping and fluorescence ISH (FISH) in BM samples. The samples were cultured as direct and overnight cultures, followed by harvesting of lymphocytes, fixation, and FISH. The FISH panel included LSI 7q31 probe, LSI IGH (14q32), LSI BCL6 (3q27), LSI c-MYC (8q24), LSI ALK (2p23), and CEP 7 and CEP 8 probes from Abbott Molecular (Abbott Park, IL, USA). It also included LSP TCRα (14q11) probe (CytoTest, Rockville, MD, USA) and LSP TCRβ (7q34) probe (Cytocell, Begbroke Science Park, Oxfordshire, UK). Two hundred interphase cells were analyzed on the Applied Spectral Imaging GENASIS software platform (Netzer Sereni, Israel) and reported according to the International System for Human Cytogenomic Nomenclature (ISCN) 2016.

## 3 Statistical Analysis

The median and range of the various parameters were evaluated using Microsoft Office Excel version 16. The relation between the type of biopsy (SEB vs. CNB) and inadequacy of diagnosis/subtyping of T-NHL was studied using Fisher’s exact test. Similarly, the relation between the IHC panel (adequate vs. inadequate) and inadequacy of diagnosis/subtyping of T-NHL was also studied using Fisher’s exact test. p-Value <0.05 was considered statistically significant.

## 4 Results

### 4.1 Patient Characteristics

The study included 232 patients diagnosed with mature T/NK-NHLs with FCI data out of 4,862 patients evaluated using FCI for lymphoma diagnosis and/or staging between 2014 and 2020 (**Figure 1**). Clinical presentation and baseline characteristics at diagnosis are given in **Table 1**. The median age of the patients was 51 years (range, 6–85 years), and M:F ratio was 2.3:1. Our data included 16 patients younger than 18 years (M:F, 13:3), including eight patients with hepatosplenic γδT-NHL (γδHSTCL), six with anaplastic large T-cell lymphoma (ALCL), one with cutaneous γδT-NHL (γδCTCL), and one with subcutaneous panniculitis-like T-cell lymphoma (SPTCL). The detailed clinical findings were available in 91% of patients. The radiological details were available in 173/232 (PET scan, 135/232; and MRI/CT/USG scan, 76/232) patients. **Figures 1A, B** describe the distribution of patients.

**Table 1:** Demographic details of the T-cell/NK-cell NHL patients.

Characteristics	AITL/FHTCL	ALCL		CTCL/MF	ATLL	PLL	PTCL-NOS	LGLL/LGLP	γδ-HSTCL	γδCTCL	NK/NK-T	SP-TCL	I-TCL (EATCL)	Inadequate subtyping	Non-neoplastic
**Number of patients (n)**	52	18		17	05	16	42	28	28	5	10	01	02	07	01
**Age,** in years															
•Median	57	32		55	47	52.5	57.5	52	24.5	45	46.5	15	46	55	36
•(Range)	(34–84)	(7–61)		(20–75)	(37–64)	(27–69)	(19–85)	(30–76)	(6–60)	(9–60)	(32–78)			(16–75)	
**M:F ratio**	2.5:1	2.6:1		1:1.2	1.5:1	1.3:1	7.4:1	2.1:1	2.5:1	4:1	1:1.5	M	M	2.5:1	F
**Lymphadenopathy**															
•n (%)	50 (96.2)	8 (77.8)		8 (47)	4 (80)	8 (50)	35 (83.3)	5 (18)	10 (35.7)	2 (40)	5 (50)	1	0	6 (85.7)	0
**Hepatosplenomegaly**															
•n (%)	10 (19.2)	5 (27.8)		3 (18)	2 (40)	5 (31.3)	13 (30.9)	3 (11)	22 (78.5)	2 (40)	1 (10)	1	0	4 (57.1)	0
**Skin lesions**															
•n (%)	2 (3.8)	2 (11.1)		17 (100)	1 (20)	1 (6.3)	7 (16.7)	3 (11)	4 (14.2)	5 (100)	1 (10)	0	0	1 (14.3)	1
**WBC**, ×10^9^/L															
•Median	8.14	14.05		18.4	16.8	49.2	7.13	10	6.68	9.17	8.02	6.1	10.4	11.1	12.3
•(Range)	(1.5–33.2)	(2.1–45.3)		(6.8–43.2)	(8–37.5)	(4–591)	(1.2–56.5)	(2–106)	(1.2–608)	(3.7–17.1)	(3.1–11.7)	–	(8.8–12.1)	(5.2–19.8)	–
**Hb**, × g/L															
•Median	109	111		115	128	109	115	120	90	118	125	131	137	106	106
•(Range)	(69–160)	(55–159)		(102–156)	(104–148)	(76–152)	(59–170)	(67–148)	(51–134)	(87–152)	(111–146)		(129–145)	(50–128)	
**PLT**, ×10^9^/L															
•Median	197	209		274	273	98	202	269.5	45	385	270.5	333	258.5	215.5	245
•(Range)	(8–492)	(64–498)		(63–565)	(123–671)	(53–331)	(26–697)	(18–585)	(9–475)	(197–749)	(137–784)	–	(209–308)	(98–432)	–
**FCI—sample type**															
**PB**, n (%)	1 (1.9)	–		10 (59)	4 (80)	11(69)	4 (9.5)	14 (50)	6 (21.4)	–	–	–	–	–	–
**TL**, Median %	75.8			20.4	59.9	77.5	30.3	70.5	49.7						
(Range %)	–			(1–91)	(1.6–73.5)	(4.07–96)	(19.9–79.7)	(11.5–87.5)	(9.24–88)						
**BM**, n (%)	39 (75)	13 (72.2)		6 (35.3)	1 (20)	4 (25)	34 (81)	14 (50)	22 (78.5)	2 (40)	6 (60)	–	2 (100)	7 (100)	–
**TL**, Median %	0.53	2.1		6.6	25.3	77.5	1.47	17.9	36.3	28.7	10.05		–	0.4	
(Range %)	(0.04–53.5)	(0.44–52.8)		(3.2–48)		(2.3–88)	(0.04–47.1)	(2.07–75.6)	(1.7–89.8)	(8.1–49.3)	(0.8–24.1)		1.57 and 3.2	(0.05–5.5)	
**FNA**, n (%)	10 (19.2)	2 (11.1)		1 (5.9)	–	1 (6.3)	3 (7.1)	–	–	3 (60)	2 (20)	1 (100)	–	–	1 (100)
**TL**, Median %	15.4	5.7		42.5		57.8	66.8			14.5	48.7	40.7			
(Range %)	(1.6–40.63)	(0.3–11.02)					(2.3–84)			(14.4–44)	(19–78.6)				
**BF**, n (%)	2 (3.84)	3 (16.7)		–	–	–	1 (2.3)	–	–	–	2 (20)	–	–	–	–
**TL**, Median %	25.2	14.9					52.4				4.15				
(Range %)	(5.3–45)	(0.1–90.1)									(0.5–7.8)				
**Type of biopsy**															
•**SEB,** n (%)	43 (83)	10(55.5)	4 (24)		2 (50)	4 (25)	23 (55)	2 (7.14)	3 (10.7)	2 (40)	3 (30)	0	–	3 (43)	0
•**CNB,** n (%)	3 (5.8)	8 (44.4)	10 (59)		2 (50)	0	14 (33.3)	3 (11)	3 (10.7)	3 (60)	7 (70)	1		0	1
**CTG** n (%)	4 (7.7)	2 (11.1)	1 (5.9)		0	9 (56.3)	6 (14.3)	4 (14.2)	14 (50)	0	1 (10)	0	0	0	0

BM, bone marrow; BF, body fluids; CNB, core-needle biopsy; CTG, cytogenetics; F, female; FCI, flow cytometric immunophenotyping; FNA, fine-needle aspiration; Hb, hemoglobin; M, male; n, number; PLT, platelets; SEB, surgical excisional biopsy; TL, tumor levels; WBC, white blood cells; AITL, angioimmunoblastic T-cell lymphoma; ALCL, anaplastic large T-cell lymphoma; ATLL, adult T-cell leukemia/lymphoma; CTCL, cutaneous T-cell lymphoma; EATCL, enteropathy-associated T-cell lymphoma; FHTCL, follicular helper type T-cell lymphoma; HSTCL, hepatosplenic T-cell NHL; I, intestinal; LGLL, large granular lymphocytic leukemia; MF, mycosis fungoides; NK/NKTCL, NK-/T-cell lymphoma; PTCL-NOS, peripheral T-cell lymphoma not otherwise specified; PLL, prolymphocytic leukemia; SPTCL, subcutaneous panniculitis-like T-cell lymphoma.

### 4.2 Flow Cytometric Immunophenotypic Findings

A total of 255 samples were received for FCI at diagnosis and/or staging from 232 patients. The samples submitted included 50 PB, 173 BM, 24 FNA, and 8 BF samples. In 23 patients, a BM sample was received in addition to PB, FNA, and BF. The frequency distribution of these patients is shown in **Figure 1B**. The angioimmunoblastic T-cell lymphoma (AITL)/follicular helper type T-cell lymphoma (FHTCL) was the commonest T-NHL (52/232, 22.4%) in our cohort. BF samples (n = 8) included 3 ALCL, 2 NK-cell lymphomas (NKCLs), 2 AITL/FHTCL, and a case of PTCL-NOS. The FCI findings were negative in 34 patients due to uninvolved BM (by FCI as well as biopsy). Of the 198 samples with FCI results, a comprehensive (primary and additional) antibody panel was performed in 165 (83.3%) samples, and only a primary antibody panel was performed in 33 (16.7%) samples due to either inadequate sample or paucicellularity. The histopathological and IHC findings were not available in 41 patients. The histopathological findings were available in a total of 157 patients, but IHC was available in 150 patients. So the results of both FCI and histopathological/IHC findings were available in a total of 150 patients (**Figure 1**).

The FCI findings are described in **Table 2**. Briefly, tumor cells in AITL/FHTCL demonstrated a very characteristic immunophenotypic signature with positive expressions of CD2, CD4, CD5, CD45, and heterogeneous CD7 but negative expressions of surface CD3, CD8, CD16, CD56, CD25, CD26, and CD30. The tumor cells also expressed one or more of CD10, CXCR5, ICOS, and PD1 wherever available. Abnormal lymphocytes in cutaneous T-cell lymphoma [CTCL; including mycosis fungoides (MF)] were characterized by CD4 restriction, variable loss of CD7 and CD26, moderate CD2, moderate-to-dim surface CD3 and CD5, and heterogeneous CD279 (PD1) but negative expressions of CD8, CD10, CD16, CD56, CD57, CD185 (CXCR5), and TCRγδ. T-cell prolymphocytic leukemia (PLL) cells were characterized by homogenously moderate CD2, CD5, and CD7, variable loss of surface CD3, moderate-to-dim CD26, heterogeneous CD25, and moderate TCL1. However, they were characterized by negative expressions of CD10, CD16, CD56, CD57, CD185 (CXCR5), CD279 (PD1), and TCRγδ as described by Matutues et al. (57, 74). All cases of PLL were positive for TCL1 expression, and a subset showed heterogeneous co-expression of CD8. Tumor cells in ALCL were large with higher forward scatter (FSC)/SSC and revealed variable loss of pan-T-cell markers (i.e., CD2, CD3, CD5, CD7, and CD4 restriction) and co-expressions of CD25 and CD26 but were negative for CD10, CD16, CD56, CD57, CD185 (CXCR5), CD279 (PD1), TCL1, and TCRγδ expressions. A subset of ALCL was positive for CD30 and ALK-1. Adult T-cell leukemia/lymphoma (ATLL) cells showed typical irregular nuclear contours and were characterized by moderate CD2, CD4, and CD5, loss of surface CD3, CD7, and CD26, and dim-to-negative CD279. However, they were characterized by negative expressions of CD10, CD16, CD56, CD57, CD279 (PD1), and TCRγδ. All cases of ATLL showed homogenous CD25 expression.

**Table 2:** Expression pattern of various markers on flow cytometric immunophenotyping (n = 196).

FCM markers	Expressionpattern	AITL/FHTCL (n = 47) No. (%)	ALCL (n = 10) No. (%)	CTCL/MF (n = 14) No. (%)	ATLL (n = 5) No. (%)	PLL (n = 16) No. (%)	PTCL-NOS (n = 27) No. (%)	LGLL/LGLP (n = 28) No. (%)	GD-HSTCL (n = 28) No. (%)	GD-CTCL (n = 5) No. (%)	NKTCL (n = 8) No. (%)	SP-TCL (n = 1) No. (%)	I-TCL (n = 1) No. (%)	Inadequate subtyping (n = 7) No. (%)
**CD2**	M/B	42 (89.36)	4 (40.00)	10 (71.43)	4 (80)	13 (81.25)	19 (70.37)	18 (64.29)	6 (21.4)	3 (60)	7 (87.5)	1 (100)	1 (100)	4 (57.14)
	D/H	2 (4.26)	2 (20.00)	1 (7.14)	1 (20)	1 (6.25)	4 (14.81)	5 (17.86)	1 (3.6)	1 (20)	–	–	–	0 (0)
	Neg	–	1 (10.00)	2 (14.29)	–	–	1 (3.70)	–	1 (3.6)	1 (20)	–	–	–	0 (0)
	ND	3 (6.38)	3 (30.00)	1 (7.14)	–	2 (12.5)	3 (11.11)	5 (17.86)	20 (71.4)	–	1 (12.5)	–	–	3 (42.86)
**CD3**	M/B	5 (10.64)	2 (20.00)	8 (57.14)	–	9 (56.25)	14 (51.85)	25 (89.29)	25 (89.3)	4 (80)	–	1 (100)	–	4 (57.14)
	D/H	7 (14.89)	2 (20.00)	4 (28.57)	4 (80)	2 (12.5)	6 (22.22)	2 (7.14)	3 (10.7)	1	–	–	–	1 (14.29)
	Neg	35 (74.47)	6 (60.00)	2 (14.29)	1 (20)	5 (31.25)	7 (25.93)	1 (3.57)	–	–	8 (100)	–	1 (100)	2 (28.57)
	ND	–	–	–	–	–	–	–	–	–	–	–	–	0 (0)
**CD4**	M/B	42 (89.36)	8 (80.00)	10 (71.43)	5 (100)	11 (68.75)	17 (62.96)	4 (14.29)	1 (3.6)	1 (20)	1 (12.5)	–	1 (100)	5 (71.43)
	D/H	2 (4.26)	2 (20.00)	2 (14.29)	–	3 (18.75)	3 (11.11)	–		–	–	–	–	1 (14.29)
	Neg	3 (6.38)	–	2 (14.29)	–	2 (12.50)	7 (25.93)	24 (85.71)	27 (96.4)	4 (80)	7 (87.5)	1 (100)	–	1 (14.29)
	ND	–	–	–	–	–	–	–		–	–	–	–	0 (0)
**CD5**	M/B	46 (97.87)	4 (40.00)	11 (78.57)	5 (100)	14 (87.50)	22 (81.48)	11 (39.30)	3 (10.7)	2 (40)	3 (37.5)	1 (100)	1 (100)	7 (100)
	D/H	1 (2.13)	3 (30.00)	2 (14.29)	–	–	3 (11.11)	12 (42.86)	3 (10.7)	–	–	–	–	0 (0)
	Neg	–	2 (20.00)	1 (7.14)	–	1 (6.25)	2 (7.41)	3 (10.71)	22 (78.6)	3 (60)	–	–	–	0 (0)
	ND	–	1 (10.00)	–	–	1 (6.25)	–	2 (7.14)		–	5 (62.5)	–	–	0 (0)
**CD7**	M/B	19 (40.43)	3 (30.00)	2 (14.29)	–	13 (81.25)	9 (33.33)	6 (21.43)	19 (67.9)	1 (20)	4 (50)	1 (100)	1 (100)	2 (28.57)
	D/H	11 (23.40)	3 (30.00)	4 (28.57)	–	3 (18.75)	6 (22.22)	21 (75.00)	8 (28.6)	1 (20)	1 (12.5)	–	–	0 (0)
	Neg	17 (36.17)	4 (40.00)	8 (57.14)	5 (100)	–	12 (44.44)	1 (3.57)	1 (3.6)	3 (60)	3 (37.5)	–	–	5 (71.43)
	ND	–	–	–	–	–	–	–		–	–	–	–	0 (0)
**CD8**	M/B	–	–	1 (7.14)	–	–	5 (18.52)	16 (57.14)	1 (3.6)	–	–	1 (100)	–	0 (0)
	D/H	2 (4.26)	1 (10.00)	–	–	6 (37.5)	3 (11.11)	8 (28.57)	9 (32.1)	2 (40)	3 (37.5)	–	–	0 (0)
	Neg	43 (91.49)	8 (80.00)	13 (92.86)	5 (100)	10 (62.50)	19 (70.37)	3 (10.71)	18 (64.3)	3 (60)	5 (62.5)	–	1 (100)	6 (85.71)
	ND	2 (4.26)	1 (10.00)	–	–	–	–	1 (3.57)		–	–	–	–	1 (14.29)
**CD10**	M/B	8 (17.02)	–	–	–	–	–	–		–	–	–	–	0 (0)
	D/H	26 (55.32)	1 (10.00)	–	–	–	4 (14.81)	–		–	–	–	–	0 (0)
	Neg	13 (27.66)	5 (50.00)	9 (64.29)	4 (80.00)	16 (100)	20 (74.07)	15 (53.57)	24 (85.7)	1 (20)	4 (50)	1 (100)	1 (100)	3 (42.86)
	ND	–	4 (40.00)	5 (35.72)	1 (20.00)	–	3 (11.11)	13 (46.43)	4 (14.3)	4 (80)	4 (50)	–	–	4 (57.14)
**CD16**	M/B	–	–	–	–	–	–	9 (32.14)	9 (32.1)	–	2 (25)	–	–	0 (0)
	D/H	–	1 (10.00)	–	–	–	1 (3.70)	9 (32.14)	2 (7.1)	–	1 (12.5)	–	–	0 (0)
	Neg	45 (95.74)	6 (60.00)	14 (100)	5 (100)	15 (93.75)	24 (88.89)	6 (21.43)	14 (50)	5 (100)	5 (62.5)	1 (100)	1 (100)	6 (85.71)
	ND	2 (4.26)	3 (30.00)	–	–	1 (6.25)	2 (7.41)	4 (14.29)	3 (10.7)	–	–	–	–	1 (14.29)
**CD25**	M/B	–	1 (10.00)	1 (7.14)	4 (80.00)	1 (6.25)	–	–	–	–	–	–	–	0 (0)
	D/H	4 (8.51)	1 (10.00)	2 (14.29)	1 (20.00)	3 (18.75)	5 (18.52)	1 (3.57)	1 (3.6)	–	1 (12.5)	1 (100)	–	0 (0)
	Neg	27 (57.45)	3 (30.00)	8 (57.14)	–	6 (37.5)	7 (25.93)	12 (42.86)	11 (39.3)	2 (40)	1 (12.5)	–	1 (100)	2 (28.57)
	ND	16 (34.04)	5 (50.00)	3 (21.43)	–	6 (37.5)	15 (55.56)	15 (53.57)	16 (57.1)	3 (60)	6 (75)	–	–	5 (71.43)
**CD26**	M/B	–	2 (20.00)	3 (21.43)	–	3 (18.75)	2 (7.41)	1 (3.57)		1 (20)	1 (12.5)	–	–	1 (14.29)
	D/H	11 (23.40)	–	3 (21.43)	1 (20.00)	6 (37.5)	7 (25.93)	3 (10.71)	1 (3.6)	2 (40)	3 (37.5)	1 (100)	–	0 (0)
	Neg	28 (59.57)	3 (30.00)	7 (50)	4 (80.00)	3 (18.75)	12 (44.44)	14 (50.00)	3 (10.7)	2 (40)	1 (12.5)	–	1 (100)	0 (0)
	ND	8 (17.02)	5 (50.00)	1 (7.14)	–	4 (20)	6 (22.22)	10 (35.71)	24 (85.7)	–	3 (37.5)	–	–	6 (85.71)
**CD30**	M/B	–	3 (30.00)	–	–	–	–	–	–	–	–	–	–	0 (0)
	D/H	–	1 (10.00)	–	–	–	–	–	–	–	–	–	–	0 (0)
	Neg	21 (44.68)	3 (30.00)	5 (35.71)	2 (40.00)	4 (25.00)	13 (48.15)	4 (14.29)	–	2 (40)	–	1 (100)	–	0 (0)
	ND	26 (55.32)	3 (30.00)	9 (64.29)	3 (60.00)	12 (75.00)	14 (51.85)	24 (85.71)	28 (100)	3 (60)	8 (100)	–	1 (100)	7 (100)
**CD 38**	M/B	1 (2.13)	–	–	–	1 (6.25)	–	3 (10.71)	10 (35.7)	2 (40)	4 (50)	1 (100)	–	0 (0)
	D/H	17 (36.17)	2 (20.00)	1 (7.14)	1 (20)	7 (43.75)	7 (25.73)	4 (14.29)	11 (39.3)	–	3 (37.5)	–	–	0 (0)
	Neg	11 (23.40)	2 (20.00)	5 (35.72)	1 (20)	7 (43.75)	8 (29.63)	5 (17.86)	3 (10.7)	1 (20)	–	–	–	2 (28.57)
	ND	18 (38.30)	6 (60.00)	8 (57.14)	3 (60.00)	1 (6.25)	12 (44.44)	16 (57.14)	4 (14.3)	2 (40)	1 (12.5)	–	1 (100)	5 (71.43)
**CD56**	M/B	–	1 (10.00)	–	–	–	–	6 (21.43)	9 (32.1)	–	7 (87.5)	–	–	0 (0)
	D/H	–	1 (10.00)	–	–	–	1 (3.70)	9 (32.14)	6 (21.4)	–	–	–	–	0 (0)
	Neg	45 (95.74)	5 (50.00)	14 (100)	5 (100)	15 (93.75)	23 (85.19)	10 (35.71)	11 (39.3)	5 (100)	1 (12.5)	1 (100)	1 (100)	6 (85.71)
	ND	2 (4.26)	3 (30.00)	–	–	1 (6.25)	3 (11.11)	4 (14.29)	2 (7.1)	–	–	–	–	1 (14.29)
**CD57**	M/B	–	–	–	–	–	–	8 (28.57)	–	–	1 (12.5)	–	–	0 (0)
	D/H	1 (2.13)	–	–	–	–	–	2 (7.14)	–	1 (20)	–	–	–	0 (0)
	Neg	1 (2.13)	–	3 (21.43)	1 (20.00)	1 (6.25)	5 (18.52)	6 (21.43)	3 (10.7)	–	3 (37.5)	1 (100)	–	0 (0)
	ND	45 (95.74)	10 (100)	11 (78.57)	4 (80.00)	15 (93.75)	22 (81.48)	12 (42.85)	25 (89.3)	4 (80)	4 (50)	–	1 (100)	7 (100)
**TCRαβ**	M/B	2 (4.26)	3 (30.00)	5 (35.72)	1 (20)	5 (31.25)	3 (11.11)	8 (28.57)		–	–	1 (100)	–	0 (0)
	D/H	–	–	–	–	–	–	–		–	–	–	–	0 (0)
	Neg	–	1 (10.00)	–	–	1 (6.25)	–	1 (3.57)	28 (100)	5 (100)	7 (87.5)	–	–	0 (0)
	ND	45 (95.74)	6 (60.00)	9 (64.29)	4 (80.00)	10 (62.50)	24 (88.89)	19 (67.86)		–	1 (12.5)	–	1 (100)	7 (100)
**TCRγδ**	M/B	–	–	–	–	–	–	2 (7.14)	25 (89.3)	4 (80)	–	–	–	0 (0)
	D/H	–	–	1 (7.14)	–	–	–	–	3 (10.7)	1 (20)	–	–	–	0 (0)
	Neg	39 (82.98)	7 (70.00)	12 (85.71)	5 (100)	14 (87.50)	26 (96.30)	22 (78.57)		–	7 (87.5)	1 (100)	1 (100)	5 (71.43)
	ND	8 (17.02)	3 (30.00)	1 (7.14)	–	2 (12.5)	1 (3.70)	4 (14.29)		–	1 (12.5)	–	–	2 (28.57)
**CD94**	M/B	–	–	–	–	–	–	2 (7.14)	2 (7.1)	1 (20)	3 (37.5)	1 (100)	–	0 (0)
	D/H	–	–	–	–	–	–	–	–	–	2 (25)	–	–	0 (0)
	Neg	1 (2.13)	1 (10.00)	1 (7.14)	–	–	2 (7.41)	5 (17.86)	–	–	1 (12.5)	–	–	0 (0)
	ND	46 (97.87)	9 (90.00)	13 (92.86)	5 (100)	16 (100)	25 (92.59)	21 (75.00)	26 (92.3)	4 (80)	2 (25)	–	1 (100)	7 (100)
**CD185 (CXCR5)**	M/B	2 (4.26)	–	–	–	–	–	–		–	–	–	–	0 (0)
	D/H	17 (36.17)	–	–	–	–	–	–		–	–	–	–	0 (0)
	Neg	9 (19.15)	1 (10.00)	3 (21.43)	1 (20.00)	1 (6.25)	7 (25.93)	3 (10.71)		1 (20)	1 (12.5)	–	1 (100)	0 (0)
	ND	19 (40.43)	9 (90.00)	11 (78.57)	4 (80.00)	15 (93.75)	20 (74.07)	25 (89.29)	28 (100)	4 (80	7 (87.5)	1 (100)	–	7 (100)
**CD279** **(PD1)**	M/B	22 (46.81)	1 (10.00)	2 (14.29)	1 (20.00)	–	2 (7.41)	–		–	–	–	–	0 (0)
	D/H	13 (27.66)	1 (10.00)	4 (28.57)	2 (40.00)	–	4 (14.81)	–		–	–	–	–	0 (0)
	Neg	4 (8.51)	–	2 (14.29)	2 (40.00)	6 (37.5)	7 (25.93)	4 (14.29)		–	–	–	1 (100)	0 (0)
	ND	8 (17.02)	8 (80.00)	6 (42.86)	–	10 (62.5)	14 (51.85)	24 (85.71)	28 (100)	5 (100)	8 (100)	1 (100)	–	7 (100)
**CD278 (ICOS)**	M/B	1 (2.13)	–	–	–	–	–	–		–	–	–	–	0 (0)
	D/H	3 (6.38)	–	–	–	–	–	–		–	–	–	–	0 (0)
	Neg	1 (2.13)	1 (10.00)	1 (7.14)	1 (20.00)	–	1 (3.70)	–		–	–	–	–	0 (0)
	ND	42 (89.36)	9 (90.00)	13 (92.86)	4 (80.00)	16 (100)	26 (96.30)	28 (100.00)	28 (100)	5 (100)	8 (100)	1 (100)	1 (100)	7 (100)
**TCL1**	M/B	2 (4.26)	–	–	–	5 (31.25)	–	–		–	–	–	–	0 (0)
	D/H	–	–	–	–	–	–	–		–	–	–	–	0 (0)
	Neg	21 (44.68)	1 (10.00)	1 (7.14)	2 (40.00)	2 (12.5)	9 (33.33)	4 (14.29)		–	–	–	–	0 (0)
	ND	24 (51.06)	9 (90.00)	13 (92.86)	3 (60.00)	9 (56.25)	18 (66.67)	24 (85.71)	28 (100)	5 (100)	8 (100)	1 (100)	1 (100)	7 (100)
**ALK1**	M/B	–	2 (20.00)	–	–	–	–	–	–	–	–	–	–	0 (0)
	D/H	–	–	–	–	–	–	–	–	–	–	–	–	0 (0)
	Neg	21 (44.68)	4 (40.00)	7 (50.00)	2 (40.00)	5 (31.25)	14 (51.85)	3 (10.71)	–	2 (40)	–	1 (100)	–	0 (0)
	ND	26 (55.32)	4 (40.00)	7 (50.00)	3 (60.00)	11 (68.75)	13 (48.15)	25 (89.29)	28 (100)	3 (60)	8 (100)	–	1 (100)	7 (100)
**Granzyme**	M/B	–	–	–	–	–	–	4 (14.29)	–	–	2 (25)	–	–	0 (0)
	D/H	–	–	–	–	–	–	3 (10.71)	–	–	–	1 (100)	–	0 (0)
	Neg	–	–	2 (14.29)	–	1 (6.25)	1 (3.70)	–	–	2 (40)	1 (12.5)	–	–	0 (0)
	ND	47 (100)	10 (100)	12 (85.71)	5 (100)	15 (93.75)	26 (96.30)	21 (75.00)	28 (100)	3 (60)	5 (62.5)	–	1 (100)	7 (100)
**Perforin**	M/B	–	–	–	–	–	–	2 (7.14)	–	–	2 (25)	–	–	0 (0)
	D/H	–	–	–	–	1 (6.25)	–	2 (7.14)	–	–	–	1 (100)	–	0 (0)
	Neg	–	–	2 (14.29)	–	–	1 (3.70)	2 (7.14)	–	2 (40)	1 (12.5)	–	–	0 (0)
	ND	47 (100)	10 (100)	12 (85.71)	5 (100)	15 (93.75)	26 (96.30)	22 (78.57)	28 (100)	3 (60)	5 (62.5)	–	1 (100)	7 (100)
**TCR-Vβ**	DC	9 (19.14)	1 (10.00)	5 (35.71)	–	6 (37.50)	7 (25.93)	17 (60.71)	–	–	1 (12.5)	–	–	2 (28.57)
	IC	–	–	3 (21.43)	3 (60.00)	–	1 (3.70)	1 (3.57)	–	–	–	1 (100)	–	0 (0)
	ND	38 (80.85)	9 (90.00)	6 (42.86)	2 (40.00)	10 (62.50)	19 (70.37)	10 (35.71)	28 (100.00)	5 (100)	7 (87.5)	–	1 (100)	5 (71.43)
**TRBC1**	Pos	1 (2.13)	–	–	–	–	1 (3.70)	2 (7.14)	–		1 (12.50)		–	0 (0)
	Neg	–	1 (10.00)	2 (14.29)	1 (20.00)	1 (6.25)	2 (7.41)	3 (10.71)						0 (0)
	ND	46 (97.87)	9 (90.00)	12 (85.71)	4 (80.00)	15 (93.75)	24 (88.89)	23 (82.14)	28 (100.00)	5 (100)	7 (87.50)	1 (100)	1 (100)	7 (100)

B, bright; D, dim; DC, direct clonality; H, heterogeneous; IC, indirect clonality; M, moderate; ND, not done; Neg, negative; Pos, positive; AITL, angioimmunoblastic T-cell lymphoma; ALCL, anaplastic large T-cell lymphoma; ATLL, adult T-cell leukemia/lymphoma; CTCL, cutaneous T-cell lymphoma; EATCL, enteropathy-associated T-cell lymphoma; FHTCL, follicular helper type T-cell lymphoma; HSTCL, hepatosplenic T-cell NHL; I, intestinal; LGLL, large granular lymphocytic leukemia; MF, mycosis fungoides; NK/NKTCL, NK-/T-cell lymphoma; PTCL-NOS, peripheral T-cell lymphoma not otherwise specified; PLL, prolymphocytic leukemia; SPTCL, subcutaneous panniculitis-like T-cell lymphoma.

Lymphoma cells in γδHSTCL showed blast-like morphology and were typically positive for moderate CD2, surface CD3, CD7, and TCRγδ, and weak-to-negative CD5. However, they were negative for CD4, CD10, CD16, and CD26. Approximately half of the γδHSTCL was positive for CD56 and showed heterogeneous expression of CD8. Contrary to γδHSTCL, tumor cells of γδCTCL were small to intermediate in size with mature nuclear chromatin. Immunophenotype of γδCTCL was similar to that of γδHSTCL except for the negative expressions of CD16 and CD56. Typical immunophenotype of large granular lymphocytic leukemia (LGLL) included positive expressions of CD2, surface CD3 and CD8 (occasionally CD4), heterogeneous CD7, and over-/under-expression of CD5 but negative for CD25, CD26, and TCRγδ. The LGLL cells demonstrated positive expressions for one or more of CD16, CD56, CD57, CD94, granzyme, and perforin. Of note, out of 28 patients categorized under LGLL/LGLP category, a concurrently existing small clonal large granular lymphocytic population (LGLP) (CD3+CD8+CD56+CD57+ T cells) was detected in patients diagnosed with primary mediastinal B-cell lymphoma (PMBCL), T-cell rich B-cell lymphoma (TCRBCL), and cutaneous ALCL. These patients were included in the LGLL/LGLP category (**Table 2**). Tumor cells in NKCL were consistently negative for CD3, CD4, and CD5 and showed moderate CD2, CD38, CD56, and CD94 and heterogeneous expressions of CD7, CD8, CD16, and CD26.

T-cell clonality by TCR-Vβ repertoire was performed in 57 patients and using TRBC1 antibody in 15 patients (**Table 2**). Among them, 47 cases showed direct clonality with a single TCR-Vβ protein restriction, and 9 showed indirect evidence of T-cell clonality as described previously (62). In one case, there was no evidence of clonality. On clonality assessment by TRBC1 expression, ten samples showed negative expression, and five showed positive expression in the abnormal T-cell population.

Thus, FCI allowed the definitive diagnosis and subtyping in 190/198 (95.45%) patients. Of the remaining eight patients, the diagnosis of T-NHL was suggested, but subtyping was not possible in seven patients (**Table 1**). FCI was falsely negative in a patient of ALCL (due to marked hemodilution of BM sample and focal tumor involvement).

### 4.3 Histopathology and Immunohistochemistry Findings

The histopathological (tissue and/or BM biopsy) findings were available in 191/232 patients. Among 191 patients, TB and BM biopsy findings were available in 138, with only TB findings in 18 and only BM biopsy findings in 35 patients. Out of a total of 156 TB samples, 100 (64.1%) were SEB samples and 56/191 (35.9%) were CNB samples. The IHC results were available in 184/191 (SEB, 100/100; CNB, 55/56; and BM, 29/35) patients. The IHC panel was adequate in 105/184 (57.1%) TB (SEB, 62/100, 62%; and CNB, 36/55, 65.5%) and 7/29 (24.1%) BM biopsy samples. The results of IHC are given in **Table 3**. Among these 184 patients with IHC results, the diagnosis and subtyping on histopathology/IHC evaluation were made in 125/184 (67.9%; 115/155 in patients with tissue biopsies and 10/29 BM biopsies) patients. These included 42 (22.8%) PTCL, 16 (8.7%) AITL/FHTCL, 15 (8.2%) ALCL, 14 (7.6%) CTCL, 12 (6.5%) γδHSTCL, 7 (3.8%) NKCL, 3 (1.6%) SPTCL, 2 (1.1%) ATLL, 2 (1.1%) LGLL, 1 (0.5%) γδCTCL, and 1 (0.5%) enteropathy-associated T-cell lymphoma (EATCL). In addition, 6/184 (3.2%) cases were diagnosed as B-NHL (diffuse large B-cell lymphoma (DLBCL), 3; low-grade B-NHL, 3), 3 (1.6%) as Hodgkin’s lymphoma (classical HL, 2; and nodular lymphocyte-predominant Hodgkin’s lymphoma (NLPHL), 1), and 1 (0.5%) as AL. Of the remaining 59/184 patients, the diagnosis of T-NHL was made, but further subtyping was not possible (i.e., inadequate subtyping) in 29/184 (15.7%), and the suspicion of T-NHL/atypical T-cell proliferation (i.e., inadequate diagnosis) was suggested in 22/184 (12%). The 8/184 (4.3%) cases were reported negative for T-cell NHL due to either scanty/non-representative tissue or low-level involvement (tumor cells <5% of all cells).

**Table 3:** Results of immunohistochemistry (IHC) after final diagnosis and subtyping of T-NHL (n = 184).

IHC markers	Expression	AITL/FHTCL (n = 49) No. (%)	ALCL (n = 18) No. (%)	CTCL/MF (n = 14) No. (%)	ATLL (n = 5) No. (%)	PLL (n = 6) No. (%)	PTCL-NOS (n = 38) No. (%)	LGLL (n = 11) No. (%)	γδ-HSTCL (n = 21) No. (%)	γδ-CTCL (n = 5) No. (%)	NK/NK-T (n = 10) No. (%)	SP-TCL (n = 1) No. (%)	I-TCL (n = 1) No. (%)	Inadequate subtyping (n = 5) No. (%)
**CD3**	Pos	44 (89.80)	8 (44.44)	14 (100)	5 (100)	6 (100)	35 (92.10)	7 (63.64)	20 (95.2)	5 (100)	9 (90.00)	1 (100)	1 (100)	4 (80)
	Neg	5 (10.20)	9 (50.00)	0 (0)	0 (0)	0 (0)	2 (5.26)	4 (36.36)	0 (0)	0 (0)	1 (10.00)	0 (0)	0 (0)	1 (20)
	ND	0 (0)	1 (5.56)	0 (0)	0 (0)	0 (0)	1 (2.63)	0 (0)	1 (4.76)	0 (0)	0 (0)	0 (0)	0 (0)	0 (0)
**CD4**	Pos	32 (65.31)	5 (27.78)	10 (71.43)	4 (80.00)	4 (66.67)	21 (55.26)	0 (0)	0 (0)	1 (20)	1 (10.00)	0 (0)	0 (0)	1 (20)
	Neg	3 (6.12)	0 (0)	2 (14.29)	0 (0)	1 (16.67)	1 (2.63)	3 (27.27)	9 (42.8)	2 (40)	5 (50.00)	1 (100)	1 (100)	0 (0)
	ND	11 (22.45)	13 (72.22)	2 (14.29)	1 (20.00)	1 (16.67)	16 (42.11)	8 (72.73)	12 (57.1)	2 (40)	4 (40.00)	0 (0)	0 (0)	4 (80)
**CD5**	Pos	21 (42.86)	2 (11.11)	8 (57.14)	2 (40.00)	2 (33.33)	13 (34.21)	0 (0)	2 (9.5)	1 (20)	1 (10.00)	0 (0)	0 (0)	1 (20)
	Neg	3 (6.12)	1 (5.56)	0 (0)	0 (0)	0 (0)	0 (0)	1 (9.10)	5 (23.8)	1 (20)	2 (20.00)	0 (0)	0 (0)	0 (0)
	ND	25 (51.02)	15 (83.33)	6 (42.86)	3 (60.00)	4 (66.67)	25 (65.79)	10 (90.91)	14 (66.7)	3 (60)	7 (70.00)	1 (100)	1 (100)	4 (80)
**CD7**	Pos	25 (51.02)	4 (22.22)	4 (28.57)	0 (0)	4 (66.67)	16 (42.10)	1 (9.10)	5 (23.8)	2 (40)	6 (60.00)	0 (0)	0 (0)	1 (20)
	Neg	14 (28.57)	2 (11.11)	7 (50.00)	3 (60.00)	1 (16.67)	10 (26.31)	3 (27.27)	3 (14.3)	1 (20)	1 (10.00)	0 (0)	0 (0)	1 (20)
	ND	10 (20.40)	12 (66.67)	3 (21.43)	2 (40.00)	1 (16.67)	12 (31.58)	7 (63.64)	13 (61.9)	2 (40)	3 (30.00)	1 (100)	1 (100)	3 (60)
**CD8**	Pos	8 (16.33)	0 (0)	0 (0)	0 (0)	1 (16.67)	3 (7.89)	2 (18.18)	2 (9.5)	2 (40)	1 (10.00)	1 (100)	1 (100)	1 (20)
	Neg	27 (55.10)	4 (22.22)	9 (64.29)	2 (40.00)	3 (50.00)	17 (44.74)	1 (9.10)	7 (33.3)	1 (20)	5 (50.00)	0 (0)	0 (0)	0 (0)
	ND	14 (28.57)	14 (77.78)	5 (35.71)	3 (60.00)	2 (33.33)	18 (47.36)	8 (72.72)	12 (57.1)	2 (40)	4 (40.00)	0 (0)	0 (0)	4 (80)
**CD10**	Pos	9 (18.36)	0 (0)	0 (0)	0 (0)	0 (0)	1 (2.63)	1 (9.10)	0 (0)	0 (0)	0 (0)	0 (0)	0 (0)	0 (0)
	Neg	15 (30.61)	2 (11.11)	1 (7.14)	3 (60.00)	2 (33.33)	9 (23.68)	2 (18.18)	3 (14.3)	0 (0)	2 (20.00)	0 (0)	0 (0)	1 (20)
	ND	25 (51.02)	16 (88.89)	13 (92.86)	2 (40.00)	4 (66.67)	30 (78.94)	8 (72.72)	18 (85.7)	5 (100)	8 (80.00)	1 (100)	1 (100)	4 (80)
**CD15**	Pos	1 (2.04)	0 (0)	0 (0)	0 (0)	0 (0)	0 (0)	0 (0)	0 (0)	0 (0)	0 (0)	0 (0)	0 (0)	0 (0)
	Neg	3 (6.12)	0 (0)	0 (0)	0 (0)	0 (0)	2 (5.26)	1 (9.10)	0 (0)	0 (0)	0 (0)	0 (0)	0 (0)	1 (20)
	ND	45 (91.84)	18 (100)	14 (100)	5 (100)	6 (100)	36 (94.74)	10 (90.91)	21 (100)	5 (100)	10 (100.00)	1 (100)	1 (100)	4 (80)
**CD30**	Pos	1 (2.04)	14 (77.78)	1 (7.14)	2 (40.00)	0 (0)	3 (7.89)	2 (18.18)	0 (0)	0 (0)	0 (0)	0 (0)	0 (0)	1 (20)
	Neg	14 (28.57)	2 (11.11)	6 (42.86)	2 (40.00)	1 (16.67)	12 (31.58)	2 (18.18)	0 (0)	1 (20)	2 (20.00)	1 (100)	0 (0)	1 (20)
	ND	34 (69.38)	2 (11.11)	7 (50.00)	1 (20.00)	5 ()	23 (60.53)	7 (63.64)	21 (100)	4 (80)	8 (80.00)	0 (0)	1 (100)	3 (60)
**CD56**	Pos	0 (0)	1 (5.56)	0 (0)	0 (0)	0 (0)	0 (0)	2 (18.18)	1 (4.76)	0 (0)	8 (80.00)	0 (0)	1 (100)	0 (0)
	Neg	2 (4.08)	1 (5.56)	3 (21.43)	2 (40.00)	0 (0)	5 (13.56)	1 (9.10)	1 (4.76)	2 (40)	1 (10.00)	1 (100)	0 (0)	1 (20)
	ND	47 (95.92)	16 (88.89)	11 (78.57)	3 (60.00)	6 (100)	33 (86.84)	8 (72.72)	19 (90.5)	3 (60)	1 (10.00)	0 (0)	0 (0)	4 (80)
**CD57**	Pos	0 (0)	0 (0)	0 (0)	0 (0)	0 (0)	1 (2.63)	0 (0)	0 (0)	0 (0)	0 (0)	0 (0)	0 (0)	0 (0)
	Neg	0 (0)	0 (0)	0 (0)	0 (0)	0 (0)	0 (0)	0 (0)	0 (0)	1 (20)	1 (10.00)	0 (0)	0 (0)	0 (0)
	ND	49 (100)	18 (100)	14 (100)	5 (100)	6 (100)	37 (97.37)	11 (100)	21 (100)	4 (80)	9 (90.00)	1 (100)	1 (100)	5 (100)
**ALK1**	Pos	0 (0)	8 (44.44)	0 (0)	0 (0)	0 (0)	0 (0)	0 (0)	0 (0)	0 (0)	0 (0)	0 (0)	0 (0)	0 (0)
	Neg	0 (0)	7 (38.89)	0 (0)	3 (60.00)	0 (0)	2 (5.26)	0 (0)	0 (0)	0 (0)	1 (10.00)	0 (0)	0 (0)	1 (20)
	ND	49 (100)	3 (16.67)	14 (100)	2 (40.00)	6 (100)	36 (94.74)	11 (100)	21 (100)	5 (100)	9 (90.00)	1 (100)	1 (100)	4 (80)
**TIA1**	Pos	0 (0)	0 (0)	0 (0)	0 (0)	0 (0)	0 (0)	0 (0)	0 (0)	0 (0)	0 (0)	0 (0)	0 (0)	0 (0)
	Neg	0 (0)	0 (0)	0 (0)	1 (20.00)	0 (0)	0 (0)	0 (0)	0 (0)	0 (0)	0 (0)	0 (0)	0 (0)	0 (0)
	ND	49 (100)	18 (100)	14 (100)	4 (80.00)	6 (100)	38 (100)	11 (100)	21 (100)	5 (100)	10 (100.00)	1 (100)	1 (100)	5 (100)
**TdT**	Pos	0 (0)	0 (0)	0 (0)	0 (0)	0 (0)	0 (0)	0 (0)	1 (4.76)	0 (0)	0 (0)	0 (0)	0 (0)	0 (0)
	Neg	3 (6.12)	1 (5.56)	0 (0)	1 (20.00)	2 (33.33)	4 (10.53)	1 (9.10)	13 (61.9)	1 (20)	3 (30.00)	0 (0)	0 (0)	0 (0)
	ND	46 (93.88)	17 (94.44)	14 (100)	4 (80.00)	4 (66.67)	34 (89.47)	10 (90.91)	7 (33.3)	4 (80)	7 (70.00)	1 (100)	1 (100)	5 (100)
**CD34**	Pos	0 (0)	0 (0)	0 (0)	0 (0)	0 (0)	0 (0)	0 (0)	1 (4.76)	0 (0)	0 (0)	0 (0)	0 (0)	0 (0)
	Neg	0 (0)	1 (5.56)	0 (0)	1 (20.00)	1 (16.67)	2 (5.26)	2 (18.18)	13 (61.9)	0 (0)	4 (40.00)	0 (0)	0 (0)	0 (0)
	ND	49 (100)	17 (94.44)	14 (100)	4 (80.00)	5 (83.33)	36 (94.74)	9 (81.82)	7 (33.3)	5 (100)	6 (60.00)	1 (100)	1 (100)	5 (100)
**LCA** **(CD45)**	Pos	0 (0)	1 (5.56)	0 (0)	0 (0)	0 (0)	3 (7.89)	1 (9.10)	0 (0)	0 (0)	2 (20.00)	0 (0)	0 (0)	0 (0)
	Neg	0 (0)	0 (0)	0 (0)	0 (0)	0 (0)	0 (0)	0 (0)	0 (0)	0 (0)	0 (0)	0 (0)	0 (0)	0 (0)
	ND	49 (100)	17 (94.44)	14 (100)	5 (100)	6 (100)	35 (92.11)	10 (90.91)	21 (100)	5 (100)	8 (80.00)	1 (100)	1 (100)	5 (100)
**BCL6**	Pos	11 (22.45)	0 (0)	0 (0)	0 (0)	0 (0)	1 (2.63)	1 (9.10)	0 (0)	0 (0)	0 (0)	0 (0)	0 (0)	0 (0)
	Neg	12 (24.49)	4 (22.22)	1 (7.14)	1 (20.00)	1 (16.67)	6 (15.79)	0 (0)	1 (4.76)	0 (0)	3 (30.00)	0 (0)	0 (0)	0 (0)
	ND	26 (53.06)	14 (77.78)	13 (92.86)	4 (80.00)	5 (83.33)	31 (81.58)	10 (90.91)	20 (95.24)	5 (100)	7 (70.00)	1 (100)	1 (100)	5 (100)
**EBV-LMP1**	Pos	4 (8.16)	0 (0)	0 (0)	0 (0)	0 (0)	1 (2.63)	0 (0)	0 (0)	0 (0)	0 (0)	0 (0)	0 (0)	0 (0)
	Neg	14 (28.57)	5 (27.78)	0 (0)	2 (40.00)	0 (0)	6 (15.79)	3 (27.27)	0 (0)	2 (40.00)	0 (0)	0 (0)	0 (0)	1 (20)
	ND	31 (63.27)	13 (72.22)	14 (100)	3 (60.00)	6 (100)	31 (81.58)	8 (72.73)	21 (100)	3 (60.00)	10 (100)	1 (100)	1 (100)	4 (80)

Markers that were negative in all samples are not included in the table.

ND, not done; Neg, negative; Pos, positive; AITL, angioimmunoblastic T-cell lymphoma; ALCL, anaplastic large T-cell lymphoma; ATLL, adult T-cell leukemia/lymphoma; CTCL, cutaneous T-cell lymphoma; EATCL, enteropathy-associated T-cell lymphoma; FHTCL, follicular helper type T-cell lymphoma; HSTCL, hepatosplenic T-cell NHL; I, intestinal; LGLL, large granular lymphocytic leukemia; MF, mycosis fungoides; NK/NKTCL, NK-/T-cell lymphoma; PTCL-NOS, peripheral T-cell lymphoma not otherwise specified; PLL, prolymphocytic leukemia; SPTCL, subcutaneous panniculitis-like T-cell lymphoma.

Further, we investigated the effect of the type of biopsy on the inadequacy of the histological diagnosis. Interestingly, the proportion of inadequate subtyping/diagnosis was higher in SEB compared to CNB (29.9% vs. 12.7%, p = 0.017). We also asked if the adequacy of the IHC panel affected the final diagnosis. As expected, the proportion of inadequate subtyping/diagnosis was relatively higher in samples with inadequate IHC (50% vs. 18.2%, p < 0.001). Thus, the inadequate IHC panel was likely to be one of the reasons for inadequate diagnosis/subtyping of T-NHL on histopathological evaluation.

### 4.4 Cytogenetic Findings

The relevant cytogenetic studies were available only in 40 (17.2%) patients, which included 16/28 (57.14%) patients with γδHSTCL revealing isochromosome 7q or del7 in 10/14, trisomy 8 in 4/14, and both in two patients. Additional 6/40 patients showed other structural abnormalities in non-γδHSTCL T-NHLs. The rest of the patients did not show any significant cytogenetic abnormality.

### 4.5 Correlation and Discrepancies Between Flow Cytometric Immunophenotyping Findings and Histopathology/Immunohistochemistry Results

**Figure 3:**
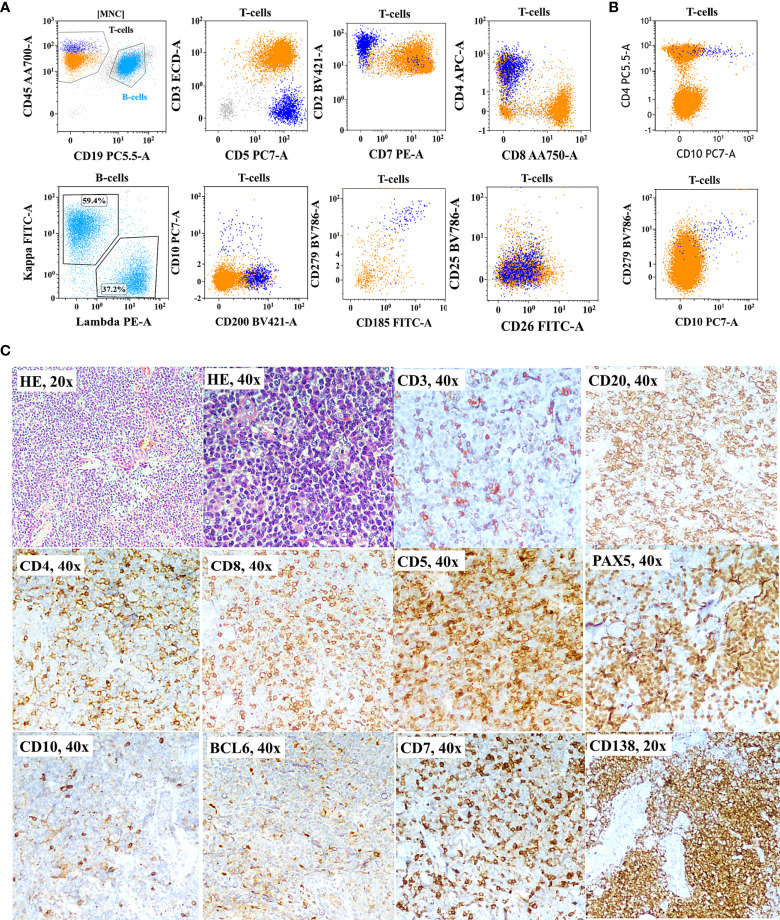
Flow cytometric immunophenotyping (FCI) findings **(A, B)** and lymph node histopathology and immunohistochemistry results **(C)** from a representative case of angioimmunoblastic T-cell-lymphoma (AITL) that was misdiagnosed as B-cell non-Hodgkin’s lymphoma (B-NHL). **(A)** The dot-plots of FCI from the lymph node fine-needle aspiration sample. **(B)** The dot-plots of FCI from the bone marrow aspiration sample from the same patient. In these dot-plots, the abnormal T cells (dark blue dots) show bright CD45, moderate CD5, bright CD2, moderate CD4, partial CD10, bright CD279 (PD1), and moderate CD185 (CXCR5) expressions but aberrant loss of surface CD3 and CD7 expressions. The orange dots represent normal T cells, and light blue dots show polyclonal B cells. **(C)** Microscopic pictures of histological (H&E staining) and immunohistochemistry results from the lymph node biopsy demonstrating B cell [highlighted by CD20 and PAX5 immunohistochemistry (IHC)] and plasma cell hyperplasia (highlighted by CD138 IHC). T cells were highlighted by CD5, CD7, CD3, CD4, and CD8 IHC.

**Figure 4:**
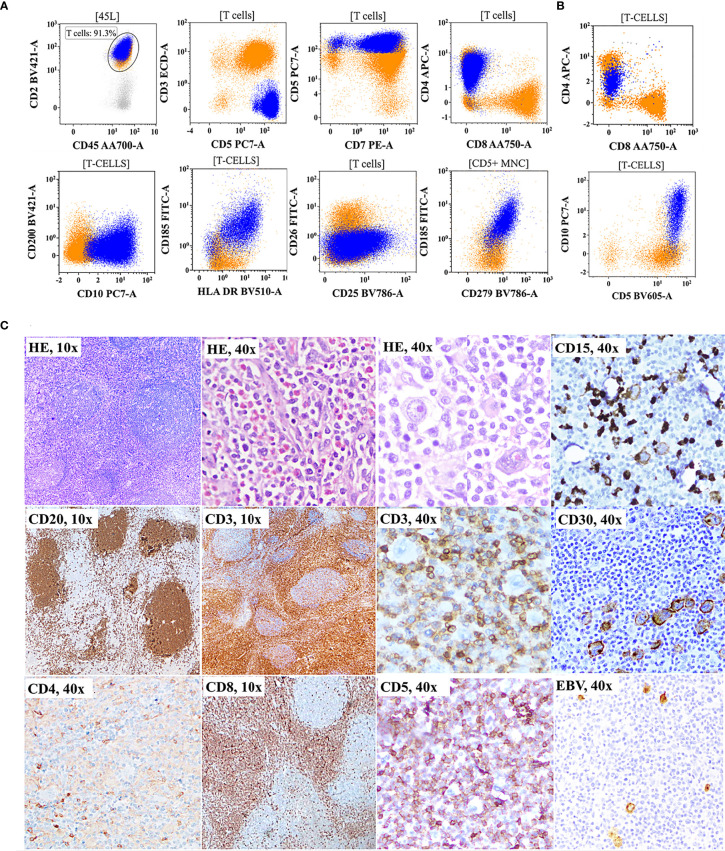
Flow cytometric immunophenotyping (FCI) findings **(A, B)** and lymph node histopathology and immunohistochemistry results **(C)** from a representative case of angioimmunoblastic T-cell-lymphoma (AITL) that was misdiagnosed as classical Hodgkin’s lymphoma (cHL). **(A)** the dot-plots of FCI from the lymph node fine-needle aspiration sample. **(B)** The dot-plots of FCI from the bone marrow aspiration sample from the same patient. In these dot-plots, the abnormal T cells (dark blue dots) show bright CD45, bright CD2, bright CD5, moderate-to-dim CD4, moderate-to-dim CD10, moderate CD279 (PD1), moderate CD185 (CXCR5), and moderate HLA-DR expressions but abnormal loss of surface CD3 expression. The orange dots represent normal T cells. **(C)** Microscopic pictures of histological (H&E staining) and immunohistochemistry results from the lymph node biopsy showing follicles highlighted by CD20 and expanded paracortex in the CD3 immunohistochemistry (IHC). Scattered Reed–Sternberg (RS)-like cells are indicated with an arrow.

**Figure 5:**
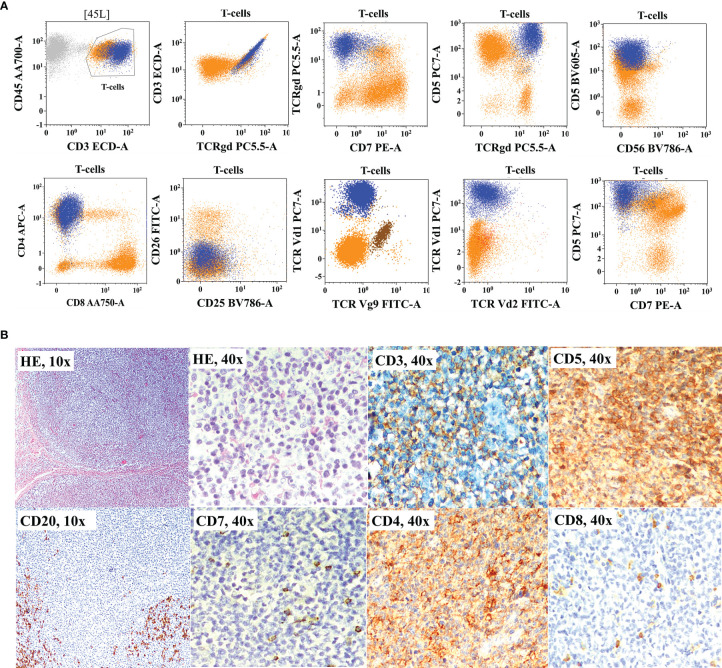
Flow cytometric immunophenotyping (FCI) findings **(A)** and histopathology and immunohistochemistry results **(B)** from a representative case of primary cutaneous γδT-cell lymphoma (γδCTCL) that was misclassified as peripheral T-cell lymphoma not otherwise classified (PTCL-NOS). **(A)** The dot-plots of FCI of fine-needle aspiration sample from the subcutaneous nodule. In these dot-plots, the abnormal γδT cells (dark blue dots) shows bright CD45, bright CD3 bright CD5, moderate CD4, and bright γδT-cell receptor (TCRγδ) expressions but abnormal loss of CD7 expression. On additional immunophenotyping, these cells also show TCRVδ1 restriction but negative TCRVδ2 and TCRVγ9 expressions. The orange dots represent normal T cells. **(B)** Microscopic pictures of histological (H&E staining) and immunohistochemistry results from the surgical biopsy from the same nodule show significantly increased proportion of CD4-positive T cells with decreased expressions of CD3 and CD7.

As shown in **Figure 1A**, correlation and discrepancies between the diagnosis using FCI and histopathological/IHC results were assessed in 150 patients. The correlation details are given in **Table 4**. A complete agreement of diagnosis and subtyping between FCI and histopathological/IHC results was found in 69/150 (46%) patients. However, of the remaining patients, 13 (of 42) patients diagnosed with PTCL-NOS on histopathology were further reclassified as AITL/FHTCL (n = 11) and T-PLL (n = 2) on FCI. These 13 patients were included in the inadequate subtyping category (**Table 4**). Additionally, FCI provided definitive diagnosis and subtyping in 25/29 patients categorized under inadequate subtyping and 21/22 patients categorized under inadequate diagnosis on histopathological results (**Table 4**). Moreover, FCI provided the correct diagnosis and subtyping in 14 patients who were misdiagnosed (6 B-NHL, 3 HL, 1 AL, and 1 SPTCL) or misclassified T-NHL (3 patients) as shown in **Table 4**. Six of nine patients misdiagnosed with B-NHL or HL were found to be involved by AITL on FCI in BM aspiration samples. Of these, 4/6 (1 DLBCL and 3 low-grade B-NHL) cases were further confirmed as AITL by additional FCI on FNA from enlarged lymph nodes, which showed abnormal T cells and polyclonal B-cell lymphocytosis (**Figure 3**). Similarly, one classical HL and one NLPHL were also re-confirmed as AITL by additional FCI on FNA from enlarged lymph nodes (**Figure 4**). Two DLBCL cases were reviewed along with follow-up biopsies and confirmed as ALCL and PTCL-NOS. One case each of classical HL, AL, PTCL-NOS, and γδHSTCL (based on sinusoidal pattern) were reclassified as PTCL-NOS, γδHSTCL, γδCTCL (**Figure 5**), and PTCL-NOS, respectively, after reviewing histopathological results along with findings of FCI. Out of the 3 SPTCL, one was reclassified as NK-/T-cell lymphoma (NKTCL), and the other case had polyclonal mixed CD4 and CD8 T-cell population on FCI in FNA from the subcutaneous nodule. The latter case was finally diagnosed as lupus erythematosus panniculitis (LEP).

**Table 4:** Correlation and discrepancies between the flow cytometric immunophenotyping (FCI) and histopathological/IHC findings. .

Type of biopsy used for diagnosis	Correlation between Histo+IHC and FCI	AITL/FHTCL n = 52	ALCL n = 18	CTCLMF n = 17	ATLL n = 5	PLL n = 16	PTCL-NOS n = 42	LGLL/LGLP n = 28	γδHSTCL n = 28	γδCTCL n = 5	NK/NKT n = 10	SP-TCL n = 1	I-TCL/EATCL n = 2	Inadequate subtyping and diagnosis by FCI n = 7	Non-neoplastic n = 1	Total n = 232	
		n (%)	n (%)	n (%)	n (%)	n (%)	n (%)	n (%)	n (%)	n (%)	n (%)	n (%)	n (%)	n (%)	n (%)	n (%)
**SEB** **(n = 100)**	**AG**	10 (19.23)	6 (33.33)	1 (5.88)	–	–	8 (19.05)	–	1 (3.57)	1 (20.00)	–	–	–	2 (28.57)	–	29 (12.5)	
	**IN-T**	15 (28.85)	1 (5.55)	2 (11.76)	3 (60.00)	3 (18.75)	1 (2.38)	1 (3.57)	1 (3.57)	1 (20.00)	-	-	-	-	-	28 (12.1)	
	**IN-D**	9 (17.31)	–	–	–	1 (6.25)	2 (4.76)	–	1 (3.57)	–	2 (20.00)	–	–	–	–	15 (6.5)	
	**MD**	5 (9.62)	-	-	-	-	1 (2.38)	-	-	-	1 (10.00)	-	-	-	1 (100	8 (3.5)	
	**CoE** **LGLP**	–	–	–	–	–	–	1 (3.57)	–	–	–	–	–	–	–	18 (7.8)	
	No FCI*	4 (7.69)	3 (16.67)	1 (5.88)	-	-	10 (23.81)	-	-	-	-	-	-	-	-	1 (0.4)	
**CNB** **(n = 55)**	**AG**	–	1 (5.55)	8 (47.06)	2 (40.00)	–	6 (14.28)	1 (3.57)	3 (10.71)	–	5 (50.00)	1 (100)	–	–	–	27 (11.6)	
	**IN-T**	1 (1.92)	1 (5.55)	-	-	-	1 (2.38)	-	-	2 (40.00)	-	-	-	-	-	5 (2.2)	
	**IN-D**	1 (1.92)	–	–	–	–	1 (2.38)	–	–	–	–	–	–	1 (14.29)	–	3 (0.9)	
	**MD**	1 (1.92)	1 (5.55)	-	-	-	2 (4.76)	-	-	1 (20.00)	-	-	-	-	-	5 (2.2)	
	**CoE** **LGLP**	–	–	–	–	–	–	1 (3.57)	–	–	–	–	–	–	–	1 (0.4)	
	No FCI*	1 (1.92)	5 (27.78)	2 (11.76)	-	-	5 (11.90)	-	-	-	2 (20.00)	-	1 (50.00)	-	-	16 (6.9)	
BM BX^**^ **(n = 36)**	**AG**	1 (1.92)	–	–	–	–	1 (2.38)	2 (7.14)	7 (25.00)	–	–	–	–	2 (28.57)	–	13 (5.6)	
	**IN-T**	1 (1.92)	-	-	-	2 (12.50)	-	1 (3.57)	5 (17.86)	-	-	-	-	-	-	9 (3.9)	
	**IN-D**	–	–	–	–	–	1 (2.38)	1 (3.57)	2 (7.14)	–	–	–	–	–	–	4 (1.7)	
	**MD**	-	-	-	-	-	-	-	1 (3.57)	-	-	-	-	-	-	1 (0.4)	
	**CoE** **LGLP**	–	–	–	–	–	–	1 (3.57)	–	–	–	–	–	–	–	1 (0.4)	
	No IHC***	2 (3.45)	-	-	-	-	1 (2.38)	1 (3.57)	2 (7.14)	-	-	-	-	1 (14.29)	-	7 (3.0)	
**No** **Histological** **Evaluation** **(n = 41)**	**-**	1 (1.92)	–	3 (17.65)	–	10 (62.50)	2 (4.76)	18 (64.3)	5 (17.86)	–	–	–	1 (50.00)	1 (14.29)	–	41 (17.7)	

AG, agreement between Histo+IHC and FCI; BM, bone marrow; Bx, biopsy; CNB, core-needle biopsy; CoE LGLP, concurrently existing clonal LGL proliferation; IN-D, inadequate diagnosis on Histo+IHC; IN-T inadequate subtyping on Histo+IHC; MD, misdiagnosis on Histo+IHC; n, number of patients; AITL, angioimmunoblastic T-cell lymphoma; ALCL, anaplastic large T-cell lymphoma; ATLL, adult T-cell leukemia/lymphoma; CTCL, cutaneous T-cell lymphoma; EATCL, enteropathy-associated T-cell lymphoma; FHTCL, follicular helper type T-cell lymphoma; HSTCL, hepatosplenic T-cell NHL; I, intestinal; LGLL, large granular lymphocytic leukemia; MF, mycosis fungoides; NK/NKTCL, NK-/T-cell lymphoma; PTCL-NOS, peripheral T-cell lymphoma not otherwise specified; PLL, prolymphocytic leukemia; SPTCL, subcutaneous panniculitis-like T-cell lymphoma.

*FCI was not available, and the diagnosis was based on histopathological/IHC and other results.

**Diagnosis was based on histopathological/IHC of BM biopsy samples only. Other tissue biopsies were not available.

***Histopathological evaluation was available, but IHC was not done.

### 4.6 Correlation and Discrepancies Between Flow Cytometric Immunophenotyping Findings and Histopathology/Immunohistochemistry Results in Bone Marrows of T-Cell Non-Hodgkin’s Lymphoma

Of the 232 patients, BM aspiration and BM biopsy samples were available in 173 patients, but IHC was done in only 83 samples. So we correlated FCI findings on BM aspiration with corresponding BM biopsy results in these 83 samples. We observed an agreement on the BM involvement by T-NHL in 50/83 (60.2%) between FCI on BM aspiration and corresponding BM biopsy findings. Among these 50, there was also an agreement on the further subtyping in 36 samples. However, histopathological/IHC evaluation of BM biopsy findings was reported suspicious for involvement by T-NHL in 7/83 (8.4%) samples and no T-NHL involvement in 27/83 (32.5%) samples, highlighting the critical value of FCI for assessment of BM samples and hence staging. Notably, in 9 of these 27 samples (33.3%), the level of tumor burden was lower than 5%, indicating the utility of high-sensitivity FCI in detecting the low-level involvement in T-NHL. FCI has also missed the BM involvement in one sample (ALCL) due to marked hemodilution and focal tumor involvement. Thus, FCI shows a distinct advantage in detecting BM involvement by T-NHLs, especially in patients with AITL and patients with low-level involvement not easily discernable by morphology.

## 5 Discussion

In the present study, we investigated the contribution of FCI in the diagnosis, classification, and staging of T- and NK-cell NHL in routine clinical practice. We retrospectively analyzed the data from 232 patients diagnosed with T/NK-NHL and correlated the results of FCI with histopathological/IHC findings in 150 patients. FCI had identified the tumor cells in 198 patients (34 were uninvolved). Although a majority of the samples for FCI were primarily submitted for staging, it successfully provided the diagnosis and subtyping of T-NHL and NK-NHL in 190/198 (96%) patients. These data suggested an invaluable role of FCI in the diagnosis and subtyping of T-NHL, especially in BM samples primarily submitted for staging purposes.

The correlation between FCI and histopathological/IHC findings (n = 150) revealed an agreement for diagnosing and subtyping in 46% of patients. However, our data showed that in more than one-third of cases (13/42) diagnosed as PTCL-NOS on histopathology, they could be further subclassified as AITL and PLL on FCI. Additionally, FCI provided the diagnosis and subclassification in those cases where adequate subtyping was not possible, and the diagnosis of T-NHL was difficult on histopathological/IHC analysis. We also studied the effect of factors such as the limited tissue availability due to CNB or limited IHC panel on the final impression of histopathological examination. As expected, the frequency of inadequate subtyping and diagnosis was relatively higher in samples where the IHC panel was limited. However, our data did not show an effect of CNB on inadequate histopathological impression. There are controversial reports on the advantages or limitations of CNB in lymphoma diagnosis. Although these studies emphasize the advantages of SEB over CNB, these reports are predominantly focused on B-NHL with a limited cohort of T-NHL (29, 30, 32, 75).

AITL was one of the commonest T-NHL (22.4%) in our cohort, which is in line with the earlier data published from India (11). It is also known for its usual presentation with advanced clinical stages involving BM and other extranodal sites (76). We diagnosed AITL in the BM samples from 41/49 (83.7%) patients studied for FCI. AITL is a well-established and relatively common subtype among the group of T-NHLs (26, 76–81). However, it is challenging to diagnose due to the lack of a unique histopathological pattern and its relatively low tumor burden in the background of abundant inflammatory cells (26, 77, 79, 81, 82). Additionally, it is characterized by reactive B-cell/plasma cell proliferations obscuring tumor cells on histopathological evaluation, which can sometimes lead to misdiagnosis of B-NHL (26, 45, 47, 76, 77, 79, 83, 84). Our data included four cases that mimicked B-NHL due to florid B-cell proliferation, which were later confirmed as AITL after demonstrating polyclonal B-cell proliferation and the presence of abnormal clonal T cells with follicular-helper T-cell immunophenotype on FCI. Occasionally, large-size immunoblasts with Reed–Sternberg (RS) cell-like appearance are seen in AITL. The presence of background cells with an admixture of reactive inflammatory cells such as eosinophils and plasma cells along with RS-like cells can mimic the morphology of HL (47, 63). We also had two cases of AITL, which were initially diagnosed as classical HL and NLPHL on histopathological evaluation and FCI on FNA, and follow-up repeat biopsy corrected the diagnosis. A reliable diagnosis of AITL requires a higher degree of suspicion and a large (and multicolor) IHC panel inclusive of immune makers specific to recognize its follicular helper T-cell origins such as CD10, CXCR5, PD1, ICOS, and CXCL13 (77, 79, 81, 82). Unfortunately, a large IHC panel in real-world practice may not be possible, especially in cases with a low degree of suspicion of T-NHL due to misleading morphology. It is also challenging to identify a small population of tumor cells in the background of reactive T cells based on the single-marker IHC. In contrast, FCI has the unique ability to identify rare tumor cells and simultaneously detect many tumor-associated molecules. An additional advantage of FCI is that it can confidently recognize even a minor alteration in the expression levels of T-cell markers such as CD3, CD5, and CD7. AITL cells are characterized by downregulation of surface CD3 and CD7 expressions, homogenous CD5, and heterogeneous CD10 expression. These markers are commonly included in the FCI panel. Hence, FCI can easily distinguish AITL tumor cells in the reactive lymphoid proliferations and helps in diagnosing AITL correctly (26, 85–87).

As shown in **Table 4**, AITL was the most common type of T-NHL inadequately subclassified on histopathological examination. Furthermore, our study included 11 cases misdiagnosed (6 B-NHL, 3 HL, 1 AL, and 1 SPTCL), and 3 cases were misclassified. Most of the cases misdiagnosed as B-NHL and HL were again confirmed as AITL on FCI in FNA and BM samples. The misclassified cases include SPTCL, γδ-TCL, and NK-TCL. Thus, FCI has a distinct role in picking up as well as preventing diagnosis in AITL, the commonest T-cell NHL in this series.

Another diagnostically challenging T-NHL on histopathological examination is γδ-TCL (γδHSTCL and γδ-CTCL) (88–91). Our study included 28 cases of γδHSTCL and 5 cases of γδ-CTCL. The γδHSTCL often presents with extranodal involvement such as in the liver, spleen, and BM (92). Hence, the diagnostic tissue is usually in the form of BM biopsy or liver/spleen CNB (88, 90). Although it is characterized by typical sinusoidal involvement, a limited tissue from BM or CNB makes it challenging to identify the scanty tumor cells and characterize them further using a limited IHC panel. Moreover, the mAb against TCRγδ for IHC was not easily available until recently. Even if available, it has limited reproducibility due to technical issues (91, 93). Furthermore, it is difficult to differentiate reactive versus abnormal γδT cells in tissues with scant involvement. In our study, histopathological findings resulted in inadequate diagnosis/subtyping of 9/28 (32%) cases of γδHSTCL, and one case each was misclassified as AL and PTCL-NOS. Alternatively, mAb against TCRγδ used in FCI is readily available and consistently reproducible. Thus, blastic morphology and FCI provide an accurate and fast diagnosis in γδHSTCL (90). Similarly, FCI in FNA samples from subcutaneous/submucosal lesions and BM samples helped in the correct diagnosis of 4 out 5 cases of γδCTCL.

Our data revealed that patients with PLL and ATLL also faced similar diagnostic dilemmas on histopathological assessment. PLL and ATLL are often diagnosed on PB smear examination and FCI, but nodal and extranodal (skin) involvement is also seen in a significant proportion of patients (14, 94). These can be easily misclassified as PTCL-NOS in the absence of IHC markers such as TCL-1 and CD25 (74). Both diseases usually present with specific immunophenotypic signatures on FCI and typical morphology on PB/BM smears. We also found 2 cases misdiagnosed as SPTCL. One was corrected as NKTCL, and the other showed normal polyclonal T cells FCI on FNA samples. The latter case was further confirmed as LEP. SPTCL is also an extremely rare T-NHL and is traditionally diagnosed on histopathological/IHC findings (95). LEP has histologically and NKTCL immunophenotypically (cytotoxic T-cell phenotype) overlapping features with SPTCL creating diagnostic dilemmas (95, 96).

Besides the availability of clinical information, histopathological findings, and immunophenotypic profile, T-cell clonality assessment is essential for diagnosing T-NHL in a substantial number of cases. T-cell clonality assessment through molecular studies is time-consuming, is less sensitive, needs additional tissue, and does not provide the immunophenotype of clonal T cells (62). In contrast, FCI allows simultaneous assessment of abnormal immunophenotype and T-cell clonality in the immunophenotypically selected suspicious population using TCR-Vβ repertoire and recently introduced a single TRBC1 antibody (71, 73, 97–99). FCI-based T-cell clonality assessment allows the detection of even a small population of clonal T cells in the background of normal T cells, thus providing a highly sensitive tool (62, 72). We studied TCR-Vβ repertoire-based T-cell clonality in 57 samples. Among them, 47 samples showed direct clonality through a single Vβ protein restriction, and nine samples showed indirect evidence of clonality through markedly reduced usage of all 24 Vβ proteins. We also used a single-marker, TRBC1, in 15 samples and found complete positive restriction in five and negative expression in ten cases, confirming the clonal proliferation of T cells. TRBC1 being a single antibody is cost-effective as compared to the TCR-Vβ repertoire. Thus, FCI provided an additional advantage of T-cell clonality assessment wherever required.

Next, we studied the correlation between FCI in BM aspiration and BM biopsy samples for the T-NHL involvement investigated as a part of clinical staging. FCI confirmed the BM involvement by T-NHL in samples with suspicion of involvement in 8.4% and no involvement in 32.5% of samples. In approximately one-third of samples where BM biopsy findings could not detect T-NHL involvement, the tumor burden was less than 5%. On the contrary, FCI missed the involvement in only one sample due to hemodilution and focal involvement. Earlier studies have also reported similar findings (87, 100). These observations highlighted the critical value of FCI for the assessment of BM involvement for correct clinical staging in T-NHL.

Overall, these data highlighted the limitations of purely histopathology-based diagnosis and subclassification. In a significant proportion of patients, the initial diagnosis and subtyping were corrected after FCI findings were incorporated as part of staging or follow-up evaluation. These results strongly argue for the simultaneous workup for histopathological evaluation, IHC, and FCI at diagnosis only. Thus, in addition to adequate clinical data, histopathological/IHC results and FCI findings play a vital complementary role for accurate diagnosis, classification, and staging in T/NK-NHL in real-world practice.

The present study included a cohort of consecutive patients in which FCI was performed as a part of routine diagnosis and staging. Our samples included mainly PB and BM samples but did not include many lymph node tissue or aspirates. Hence, these data do not represent all cases of T/NK-NHL diagnosed in our institution, as FCI was unavailable in all patients. Also, a proportion of T/NK-NHLs does not usually show BM involvement. Hence, the frequencies of T-NHL subtypes and other demographic parameters documented in these data may not be entirely representative. Nevertheless, our data provide a strong rationale for a prospective study with simultaneous assessment of nodal/extranodal tissues and BM samples using FCI and histopathological/IHC assessments. In conclusion, this retrospective study of the clinical impact of FCI in the correct diagnosis and subtyping of T- and NK-NHL in real-world practice demonstrates that a comprehensive FCI is a robust tool for identifying and immunophenotypic characterization of the abnormal T-cell population, even in samples with a low disease burden. We showed that FCI improves the subtyping and provides confirmatory evidence for T-NHL diagnosis. Our data also demonstrate that AITL is most frequently misdiagnosed on conventional histopathology/IHC evaluation and is the commonest T-NHL involving BM at a low level, not discernable by morphology but detectable by FCI. Thus, FCI plays a critical role in the T- and NK-cell NHL diagnosis, subtyping, and staging in real-world practice.

## Data Availability Statement

The original contributions presented in the study are included in the article/**Supplementary Material**. Further inquiries can be directed to the corresponding authors.

## Ethics Statement

The studies involving human participants were reviewed and approved by Institutional Ethical Committee, Tata Memorial Centre. Written informed consent from the participants’ legal guardian/next of kin was not required to participate in this study in accordance with the national legislation and the institutional requirements.

## Author Contributions

PT designed and performed the study, performed the data analysis, interpreted the data, performed the statistical analysis, and wrote the paper. GC performed the study, performed the data analysis, and helped with additional experiments and manuscript writing. PT, AC, PS, GC, NP, and SR performed the flow cytometric immunophenotyping and analyzed the data. SGG, KG, and NDe performed the quality control of the study and processed the samples for flow cytometry. AC, NDa, TK, and SV collected the data and performed the data analysis. DS performed the cytogenetic study. TA, SG, TS, and SE performed the histopathological evaluation. MS, BB, and HJ treated the patients and provided the clinical data. All authors contributed to the manuscript writing and approved the final version of the manuscript.

## Conflict of Interest

The authors declare that the research was conducted in the absence of any commercial or financial relationships that could be construed as a potential conflict of interest.

## Publisher’s Note

All claims expressed in this article are solely those of the authors and do not necessarily represent those of their affiliated organizations, or those of the publisher, the editors and the reviewers. Any product that may be evaluated in this article, or claim that may be made by its manufacturer, is not guaranteed or endorsed by the publisher.
