# Supplementary figures and images for: Critical Role of Flow Cytometric Immunophenotyping in the Diagnosis, Subtyping, and Staging of T-Cell/NK-Cell Non-Hodgkin’s Lymphoma in Real-World Practice: A Study of 232 Cases From a Tertiary Cancer Center in India
Source: Front Oncol. 2022 Mar 1;12:779230. doi: 10.3389/fonc.2022.779230 (PMC8923658; doi:10.3389/fonc.2022.779230)

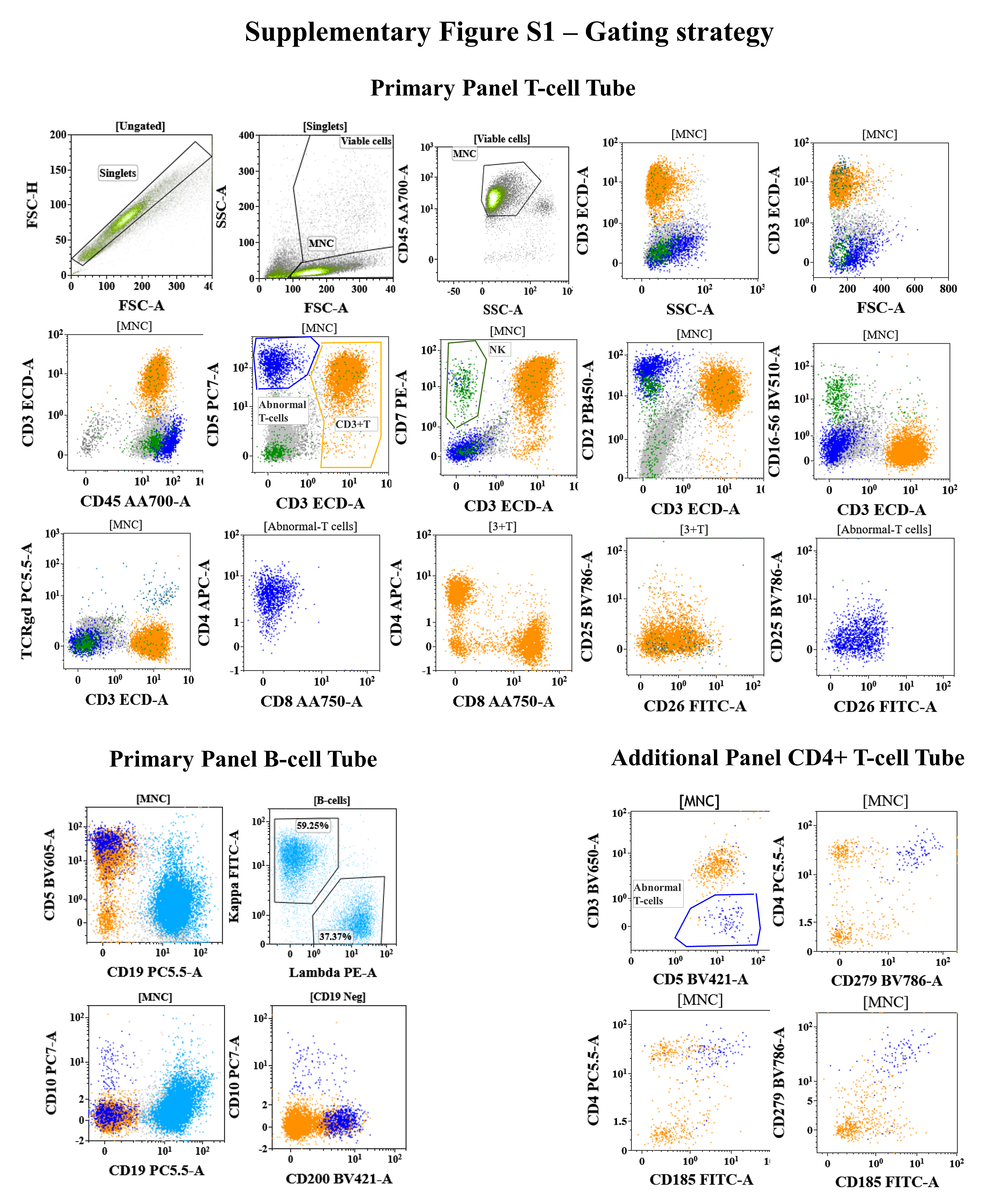

Supplement: Supplementary file 1 [file Image_1.tif]
