# Supplementary material for: Critical Role of Flow Cytometric Immunophenotyping in the Diagnosis, Subtyping, and Staging of T-Cell/NK-Cell Non-Hodgkin’s Lymphoma in Real-World Practice: A Study of 232 Cases From a Tertiary Cancer Center in India
Source: Front Oncol. 2022 Mar 1;12:779230. doi: 10.3389/fonc.2022.779230 (PMC8923658; doi:10.3389/fonc.2022.779230)
Supplement: Supplementary file 2 [file Table_1.docx]

**Supplementary Table -S1. The details of immunohistochemistry markers.**

| **Antibody** | **Protocol** | **Dilution** | **Clone** | **Vendor** |
| --- | --- | --- | --- | --- |
| CD1a | STD56 | 1;200 | EP3622 | Cell Marque |
| CD3 | Mild 1hr | 1;400 | POLY | DAKO |
| CD4 | STD 1hr | 1;100 | SP35 | Cell Marque |
| CD5 | STD 36hr | 1;400 | 4C7 | DAKO |
| CD7 | STD 60hr | 1;700 | MRQ56 | Cell Marque |
| CD8 | STD 16hr | RTU | SP57 | Roche |
| CD10 | STD 1.32hr | RTU | SP67 | Roche |
| CD15 | STD 1hr | 1;200 | carb1 | DAKO |
| CD20 | Mild 32hr | 1;400 | l26 | DAKO |
| CD23 | STD1hr | 1;400 | 1B12 | Biocare |
| CD30 | STD 1.30hr | 1;150 | Ber-H2 | DAKO |
| CD31 | Mild 32hr | 1;1000 | JC70A | DAKO |
| CD34 | STD 56 | 1;200 | QBEnd10 | DAKO |
| CD43 | STD 32hr | 1;100 | FDP1 | DAKO |
| CD56 | STD 56 | 1;400 | BC56C04 | DBS |
| CD57 | STD 32hr | 1;200 | TB01 | DAKO |
| CD138 | STD 44 | 1;250 | M115 | DAKO |
| CD163 | STD 32hr | 1;100 | 10D6 | Neobio |
| Alk1 | STD 1hr | 1;50 | ALK1 | Cell Marque |
| TdT | STD 40 | 1;100 | POLY | Cell Marque |
| LCA | Mild 32hr | 1;300 | 2b11+PD7/26 | DAKO |
| MPO | Short 32 | 1;1800 | POLY | DAKO |
| BCL2 | STD 52 | 1;300 | 12'4 | Cell Marque |
| BCL6 | STD 56 | 1;100 | G1191E/A8 | Cell Marque |
| EBV-LMP1 | Mild8 | 1;800 | CS.1-4 | DAKO |
| MUM1 | STD 32hr | 1;200 | MRQ43 | Cell Marque |
| PAX-5 | STD 32hr | 1;100 | DAK-PAX5 | DAKO |
| EMA | Mild 44 | 1;150 | E29 | DAKO |
| AE1/AE3 | STD 32hr | 1;300 | Aε1Aε3 | Biocare |
| BOB-1 | STD 32hr | 1;200 | SP92 | Cell Marque |
| Oct-02 | STD 1hr | 1;100 | MRQ-2 | Cell Marque |
| CK7 | STD 32hr | 1;500 | OVT12/30 | DAKO |
| CK20 | STD 32hr | 1;150 | KS20.8 | DAKO |
| MIB1 | STD 32hr | 1;400 | MiB-1 | DAKP |
| C-Kit | STD 1hr | 1;1000 | PLOY | DAKO |
| Pax8 | STD 1hr | 1;500 | MRQ-50 | Cell Marque |
| C-MYC | STD 1hr | 1;100 | EP121 | Cell Marque |
| TIA1 | STD 1hr | 1;100 | CM130B | Biocare |
