# Supplementary material for: Critical Role of Flow Cytometric Immunophenotyping in the Diagnosis, Subtyping, and Staging of T-Cell/NK-Cell Non-Hodgkin’s Lymphoma in Real-World Practice: A Study of 232 Cases From a Tertiary Cancer Center in India
Source: Front Oncol. 2022 Mar 1;12:779230. doi: 10.3389/fonc.2022.779230 (PMC8923658; doi:10.3389/fonc.2022.779230)
Supplement: Supplementary file 3 [file Table_2.docx]

**Supplementary Table S2A. A primary antibody panel for FCI of NHL.**

| **Fluoro-chromes** | **BV**  **786** | **BV**  **650** | **BV**  **605** | **BV**  **510** | **BV**  **421** | **FITC** | **PE** | **ECD** | **PC5.5** | **PC7** | **APC** | **APC-AF700** | **APC – AF750** |  |
| --- | --- | --- | --- | --- | --- | --- | --- | --- | --- | --- | --- | --- | --- | --- |
| **B-cell**  **Panel** | **x** | **CD11c** | **CD5** | **CD20** | **CD200** | **Kappa** | **Lambda** | **CD49d** | **CD19** | **CD10** | **CD23** | **CD45** | **CD38** |  |
| *Clone* |  | *B-ly6* | *L17F12* | *2H7* | *MRC OX-104* | *Polyclonal* | *Polyclonal* | *9F10* | *J3-119* | *ALB1* | *M-L233* | *J.33* | *LS198-4-3* |  |
| *Company* |  | *BD* | *BL* | *BD* | *BD* | *BC* | *BC* | *BD* | *BC* | *BC* | *BD* | *BC* | *BC* |  |
| **T-cell**  **Panel** | **CD25** | **x** | **x** | **CD16 + CD56** | **CD2** | **CD26** | **CD7** | **CD3** | **TCR γδ** | **CD5** | **CD4** | **CD45** | **CD8** |  |
| *Clone* | *M-A251* |  |  | *3G8/ NCAM16.2* | *RPA-2.10* | *L272* | *8H8.1* | *UCHT1* | *IMMU510* | *BL1a* | *13B8.2* | *J.33* | *B9.11* |  |
| *Company* | *BD* |  |  | *BD/BD* | *BD* | *BD* | *BC* | *BC* | *BC* | *BC* | *BC* | *BC* | *BC* |  |
| ***Abbreviations:*** BC, Beckman Coulter; BD, Becton Dickinson biosciences; BL, BioLegend; MB, Miltenyi Biotech, | | | | | | | | | | | | | | |

**Supplementary Table S2B. An additional antibody panel for FCI of NHL.**

| **A 13-color antibody panel for CD4+ T-NHL.** | | | | | | | | | | | | | | |
| --- | --- | --- | --- | --- | --- | --- | --- | --- | --- | --- | --- | --- | --- | --- |
| **Fluoro-chromes** | **BV**  **786** | **BV**  **650** | **BV**  **605** | **BV**  **510** | **BV**  **421** | **FITC** | **PE** | **ECD** | **PC5.5** | **PC7** | **APC** | **APC-AF700** | **APC – AF750** |  |
| **Tube-1** | **CD25** | **CD3** | **CD5** | **CD4** | **CD8** | **CD30** | **ALK-1** | **CD7** | **CD33** | **CD2** | **CD57** | **CD45** | **CD38** |  |
| *Clone* | *M-A251* | *UCHT1* | *L17F12* | *SK3* | *B9.11* | *KI2* | *ALK1* | *M-T701* | *D3HL60.251* | *39C1.5* | *TB03* | *J.33* | *LS198-4-3* |  |
| *Company* | *BD* | *BL* | *BL* | *BD* | *BC* | *MB* | *BD* | *BD* | *BC* | *BC* | *MB* | *BC* | *BC* |  |
| **Tube-2** | **CD279** | **CD3** | **CD5** | **CD4** | **CD8** | **CD185** | **TCL-1** | **CD7** | **CD33** | **CD2** | **HLA DR** | **CD45** | **CD278** |  |
| *Clone* | *EH12.1* | *UCHT1* | *L17F12* | *SK3* | *B9.11* | *RF8B2* | *Jan-21* | *M-T701* | *D3HL60.251* | *39C1.5* | *G46-6* | *J.33* | *REA192* |  |
| *Company* | *BD* | *BL* | *BL* | *BD* | *BC* | *BD* | *BL* | *BD* | *BC* | *BC* | *BD* | *BC* | *MB* |  |
| **A 13-color antibody panel for CD8+ T-NHL.** | | | | | | | | | | | | | | |
| **Tube-1** | **CD16** | **CD3** | **CD5** | **CD56** | **CD161** | **CD94** | **CD7** | **CD38** | **CD4** | **CD244** | **CD57** | **CD45** | **CD8** |  |
| *Clone* | *3G8* | *UCHT1* | *L17F12* | *NCAM16.2* | *DX12* | *3B2/TA8* | *8H8.1* | *LS198.4.3* | *13 B8.2* | *C1.7* | *TB03* | *J.33* | *B9.11* |  |
| *Company* | *BD* | *BL* | *BL* | *BD* | *BD* | *MB* | *BC* | *BC* | *BC* | *BL* | *MB* | *BC* | *BC* |  |
| **Tube-2** | **CD56** | **CD3** | **CD5** | **CD56** | **CD7** | **GRANZYME B** | **PERFORIN** | **CD38** | **TCR γδ** | **x** | **CD57** | **CD45** | **CD8** |  |
| *Clone* | *NCAM16.2* | *UCHT1* | *L17F12* | *NCAM16.2* | *M-T701* | *REA226* | *delta G9* | *LS198.4.3* | *IMMU510* |  | *TB03* | *J.33* | *B9.11* |  |
| *Company* | *BD* | *BL* | *BL* | *BD* | *BD* | *MB* | *MB* | *BC* | *BC* |  | *MB* | *BC* | *BC* |  |

**Supplementary Table S2C. An additional antibody panel of TCRVβ repertoire for T-cell clonality assessment by FCI.**

| **Fluoro-chromes** | **BV**  **510** | **BV**  **421** | **FITC** | **PE** | **ECD** | **PC5.5** | **PC7** | **APC** | **APC-AF700** | **APC – AF750** |  |
| --- | --- | --- | --- | --- | --- | --- | --- | --- | --- | --- | --- |
| **V-beta A** | **CD4** | **CD19** | **Vb3 + Vb7.1** | **Vb5.3 + Vb7.1** | **CD3** | **TCR γδ** | **CD16+56** | **CD7** | **CD45** | **CD8** |  |
| *Clone* | *SK3* | *HIB19* | *CH92 + ZOE* | *3D11 + ZOE* | *UCHT1* | *IMMU510* | *3G8/N901* | *8H8.1* | *J.33* | *B9.11* |  |
| *Company* | *BD* | *BD* | *BC* | *BC* | *BC* | *BC* | *BC/BC* | *BC* | *BC* | *BC* |  |
| **V-beta B** | **CD4** | **CD19** | **Vb16 +Vb17** | **Vb9 +Vb17** | **CD3** | **TCR γδ** | **CD16+56** | **CD7** | **CD45** | **CD8** |  |
| *Clone* | *SK3* | *HIB19* | *TAMAYA1.2 + E17.5F3* | *FIN9 + E17.5F3* | *UCHT1* | *IMMU510* | *3G8/N901* | *8H8.1* | *J.33* | *B9.11* |  |
| *Company* | *BD* | *BD* | *BC* | *BC* | *BC* | *BC* | *BC/BC* | *BC* | *BC* | *BC* |  |
| **V-beta C** | **CD4** | **CD19** | **Vb20 + Vb5.1** | **Vb18 + Vb5.1** | **CD3** | **TCR γδ** | **CD16+56** | **CD7** | **CD45** | **CD8** |  |
| *Clone* | *SK3* | *HIB19* | *ELL1.4 + IMMU157* | *BA62.6 + IMMU157* | *UCHT1* | *IMMU510* | *3G8/N901* | *8H8.1* | *J.33* | *B9.11* |  |
| *Company* | *BD* | *BD* | *BC* | *BC* | *BC* | *BC* | *BC/BC* | *BC* | *BC* | *BC* |  |
| **V-beta D** | **CD4** | **CD19** | **Vb8 + Vb13.6** | **Vb13.1 + Vb13.6** | **CD3** | **TCR γδ** | **CD16+56** | **CD7** | **CD45** | **CD8** |  |
| *Clone* | *SK3* | *HIB19* | *56C5.2 + JU74.3* | *IMMU222 + JU74.3* | *UCHT1* | *IMMU510* | *3G8/N901* | *8H8.1* | *J.33* | *B9.11* |  |
| *Company* | *BD* | *BD* | *BC* | *BC* | *BC* | *BC* | *BC/BC* | *BC* | *BC* | *BC* |  |
| **V-beta E** | **CD4** | **CD19** | **Vb12 + Vb2** | **Vb5.2 + Vb2** | **CD3** | **TCR γδ** | **CD16+56** | **CD7** | **CD45** | **CD8** |  |
| *Clone* | *SK3* | *HIB19* | *VER2.32 + MPB2D5* | *36213 + MPB2D5* | *UCHT1* | *IMMU510* | *3G8/N901* | *8H8.1* | *J.33* | *B9.11* |  |
| *Company* | *BD* | *BD* | *BC* | *BC* | *BC* | *BC* | *BC/BC* | *BC* | *BC* | *BC* |  |
| **V-beta F** | **CD4** | **CD19** | **Vb21.3 + Vb1** | **Vb23 + Vb1** | **CD3** | **TCR γδ** | **CD16+56** | **CD7** | **CD45** | **CD8** |  |
| *Clone* | *SK3* | *HIB19* | *IG125 + BL37.2* | *AF23 + BL37.2* | *UCHT1* | *IMMU510* | *3G8/N901* | *8H8.1* | *J.33* | *B9.11* |  |
| *Company* | *BD* | *BD* | *BC* | *BC* | *BC* | *BC* | *BC/BC* | *BC* | *BC* | *BC* |  |
| **V-beta G** | **CD4** | **CD19** | **Vb14 + Vb22** | **Vb11 + Vb22** | **CD3** | **TCR γδ** | **CD16+56** | **CD7** | **CD45** | **CD8** |  |
| *Clone* | *SK3* | *HIB19* | *CAS1.1.3 + IMMU546* | *C21 + IMMU546* | *UCHT1* | *IMMU510* | *3G8/N901* | *8H8.1* | *J.33* | *B9.11* |  |
| *Company* | *BD* | *BD* | *BC* | *BC* | *BC* | *BC* | *BC/BC* | *BC* | *BC* | *BC* |  |
| **V-beta H** | **CD4** | **CD19** | **Vb7.2 + Vb4** | **Vb13.2 + Vb4** | **CD3** | **TCR γδ** | **CD16+56** | **CD7** | **CD45** | **CD8** |  |
| *Clone* | *SK3* | *HIB19* | *ZIZOU4 + WJF24* | *H132 + WJF24* | *UCHT1* | *IMMU510* | *3G8/N901* | *8H8.1* | *J.33* | *B9.11* |  |
| *Company* | *BD* | *BD* | *BC* | *BC* | *BC* | *BC* | *BC/BC* | *BC* | *BC* | *BC* |  |
| ***Abbreviations:*** BC, Beckman Coulter; BD, Becton Dickinson biosciences; BL, BioLegend; MB, Miltenyi Biotec, | | | | | | | | | | | |

| **Supplementary Table S2D. A 13-color antibody panel for TRBC1 expression assessment.** | | | | | | | | | | | | | | |
| --- | --- | --- | --- | --- | --- | --- | --- | --- | --- | --- | --- | --- | --- | --- |
| **Fluoro-chromes** | **BV**  **786** | **BV**  **650** | **BV**  **605** | **BV**  **510** | **BV**  **421** | **FITC** | **PE** | **ECD** | **PC5.5** | **PC7** | **APC** | **APC-AF700** | **APC – AF750** |  |
| **Tube-1** | **CD56** | **CD3** | **CD5** | **CD16** | **CD4** | **CD26** | **TRBC1** | **CD7** | **CD4** | **CD2** | **CD57** | **CD45** | **CD38** |  |
| *Clone* | *NCAM16.2* | *UCHT1* | *L17F12* | *3G8* | *SK3* | *L272* | *Jovi-1* | *M-T701* | *13 B8.2* | *39C1.5* | *TB03* | *J.33* | *LS198-4-3* |  |
| *Company* | *BD* | *BL* | *BL* | *BD* | *BD* | *BD* | *LB* | *BD* | *BC* | *BC* | *MB* | *BC* | *BC* |  |
|  | | | | | | | | | | | | | | |
| **Supplementary Table S2E. A 13-color antibody panel for γδT-NHL.** | | | | | | | | | | | | | | |
| **Tube-1** | **CD56** | **X** | **CD5** | **CD16** | **CD4** | **TCRVγ9** | **CD7** | **CD3** | **TCRγδ** | **TCR Vδ1** | **CD57** | **CD45** | **CD8** |  |
| *Clone* | *NCAM16.2* | **X** | *L17F12* | *3G8* | *SK3* | *IMMU360* | *8H8.1* | *UCHT1* | *IMMU510* | *R9.12* | *TB03* | *J.33* | *B9.11* |  |
| *Company* | *BD* | **X** | *BL* | *BD* | *BD* | *BC* | *BC* | *BC* | *BC* | *BC* | *MB* | *BC* | *BC* |  |
| **Tube-2** | **CD56** | **X** | **CD5** | **CD16** | **CD4** | **TCRVδ2** | **CD7** | **CD3** | **TCRγδ** | **TCR Vδ1** | **CD57** | **CD45** | **CD8** |  |
| *Clone* | *NCAM16.2* | **X** | *L17F12* | *3G8* | *SK3* | *IMMU389* | *8H8.1* | *UCHT1* | *IMMU510* | *R9.12* | *TB03* | *J.33* | *B9.11* |  |
| *Company* | *BD* | **X** | *BL* | *BD* | *BD* | *BC* | *BC* | *BC* | *BC* | *BC* | *MB* | *BC* | *BC* |  |
